# Lactate reprograms glioblastoma immunity through CBX3-regulated histone lactylation

**DOI:** 10.1172/JCI176851

**Published:** 2024-11-15

**Authors:** Shuai Wang, Tengfei Huang, Qiulian Wu, Huairui Yuan, Xujia Wu, Fanen Yuan, Tingting Duan, Suchet Taori, Yingming Zhao, Nathaniel W. Snyder, Dimitris G. Placantonakis, Jeremy N. Rich

**Affiliations:** 1 Hillman Cancer Center and Department of Neurology, University of Pittsburgh Medical Center, Pittsburgh, Pennsylvania, USA.; 2 Department of Neurosurgery and Perlmutter Cancer Center, NYU Grossman School of Medicine, New York, New York, USA.; 3 School of Medicine, University of Pittsburgh Medical Center, Pittsburgh, Pennsylvania, USA.; 4 Ben May Department for Cancer Research, The University of Chicago, Chicago, Illinois, USA.; 5 Department of Cardiovascular Sciences, Lewis Katz School of Medicine at Temple University, Philadelphia, Pennsylvania, USA.

**Keywords:** Metabolism, Oncology, Adult stem cells, Brain cancer, Epigenetics

## Abstract

Glioblastoma (GBM), an aggressive brain malignancy with a cellular hierarchy dominated by GBM stem cells (GSCs), evades antitumor immunity through mechanisms that remain incompletely understood. Like most cancers, GBMs undergo metabolic reprogramming toward glycolysis to generate lactate. Here, we show that lactate production by patient-derived GSCs and microglia/macrophages induces tumor cell epigenetic reprogramming through histone lactylation, an activating modification that leads to immunosuppressive transcriptional programs and suppression of phagocytosis via transcriptional upregulation of *CD47*, a “don’t eat me” signal, in GBM cells. Leveraging these findings, pharmacologic targeting of lactate production augments efficacy of anti-CD47 therapy. Mechanistically, lactylated histone interacts with the heterochromatin component chromobox protein homolog 3 (CBX3). Although CBX3 does not possess direct lactyltransferase activity, CBX3 binds histone acetyltransferase (HAT) EP300 to induce increased EP300 substrate specificity toward lactyl-CoA and a transcriptional shift toward an immunosuppressive cytokine profile. Targeting CBX3 inhibits tumor growth by both tumor cell–intrinsic mechanisms and increased tumor cell phagocytosis. Collectively, these results suggest that lactate mediates metabolism-induced epigenetic reprogramming in GBM that contributes to CD47-dependent immune evasion, which can be leveraged to augment efficacy of immuno-oncology therapies.

## Abstract 2

Lactate induces epigenetic changes through histone lactylation in stem-like tumor cells to generate an immunosuppressive microenvironment. Targeting lactate generation augments efficacy of anti-tumor immunotherapy.

## Graphical abstract

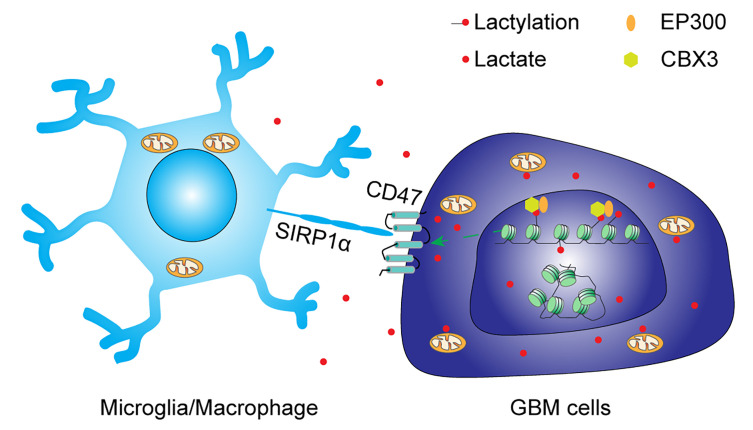

## Introduction

GBM is the most prevalent and malignant primary brain tumor in adults, with a median survival of 15–21 months despite aggressive therapy consisting of maximal safe surgical resection, radiotherapy, and temozolomide (TMZ) chemotherapy (1). GBM stem cells (GSCs) reside atop the cellular hierarchy of GBM and contribute to tumor growth, brain invasion, radioresistance, metabolic adaptations, and evasion of immune surveillance (2–4). GSCs are enriched in perivascular and perinecrotic niches (5, 6). Perinecrotic regions are characterized by hypoxia, acidic conditions, and increased inflammation. Despite the presence of immune cells, GBM cells in necrotic regions evade clearance by phagocytes, including microglia and macrophages, through multiple mechanisms, including M2 polarization of phagocytes (7) and interaction between the “don’t eat me” CD47 membrane protein on tumor cells and signal regulatory protein α (SIRPα) on phagocytes (8). Targeting interactions between GBM cells and tumor-associated microglia/macrophages may therefore promote antitumor immune responses by increasing phagocytosis (9, 10).

Tumor cell metabolism has reemerged as a prominent focus in cancer initiation and progression. In a subset of gliomas, mutations in the isocitrate dehydrogenase (IDH) enzymes act as tumor drivers, suggesting that dysregulation of metabolism promotes tumorigenesis (11). In *IDH*-WT glioblastoma (GBM), tumor metabolism is complex and its connection to tumor progression is an area of active investigation. Warburg showed that cancers metabolically shift from oxidative phosphorylation to glycolysis (12), leading to accumulation of lactate. GSCs display metabolic plasticity (13), suggesting that the regulation of glycolysis and lactate production may contribute to GSC maintenance. Dichloroacetate (DCA), an inhibitor of pyruvate dehydrogenase kinase II, which promotes production of lactate and is highly expressed in GBM, targeted GSCs with antitumor activity in a clinical trial (14). Lactate promotes tumor growth through tumor cell metabolism and epigenetic reprogramming by histone acetylation (15) and remodels the tumor microenvironment by biasing microglia polarization toward an M2-like phenotype (16). Further, the acidic microenvironment with elevated production of lactate is associated with decreased phagocytosis in GBM, although the underlying mechanism is still unclear (17). In addition to effects on innate immunity, lactate promotes the immunosuppressive microenvironment in GBM by decreasing cytotoxic T cell infiltration, proliferation, cytokine production, and antitumor cytotoxicity (18, 19). Thus, lactic acid contributes to tumor cell evasion of immune responses.

The epigenetic actions of lactate extend beyond its serving as carbon skeleton for production of acetyl-CoA, which is then utilized for histone acetylation (15, 20). Lactyl-CoA can be utilized by histone acetyltransferase (HAT) EP300 to lactylate histones during macrophage polarization (21). Lactylation of lysine residues occurs not only on histones, but also on nonhistone proteins, such as METTL3, the writer of the RNA m6A modification, which promotes immunosuppression of tumor-infiltrating myeloid cells in colon cancer (22), and tumor suppressor TP53 (23). From the functional perspective, protein lactylation not only influences epigenetic regulation through histone modifications, but also modulates cellular epitranscriptomics. Besides the lactylation of m^6^A writer METTL3 (22), histone lactylation induces expression of the reader of the m^6^A RNA modification, YTH N6-methyladenosine RNA binding protein F2 (YTHDF2), in ocular melanoma (24). Together, epigenetic and epitranscriptomic influences of lactate through protein lactylation contribute to tumor growth and immunosuppression. However, the mechanisms and phenotypic effects of protein lactylation in GBM biology are poorly understood. Here, we investigate the role of lactate in regulating the epigenetic and immunologic landscapes in GBM. Our results suggest that histone lactylation promotes tumor growth through direct effects on tumor cells and suppression of their phagocytosis by microglia and macrophages.

## Results

### GSCs display high levels of histone lactylation augmented by microglia.

**Figure 1:**
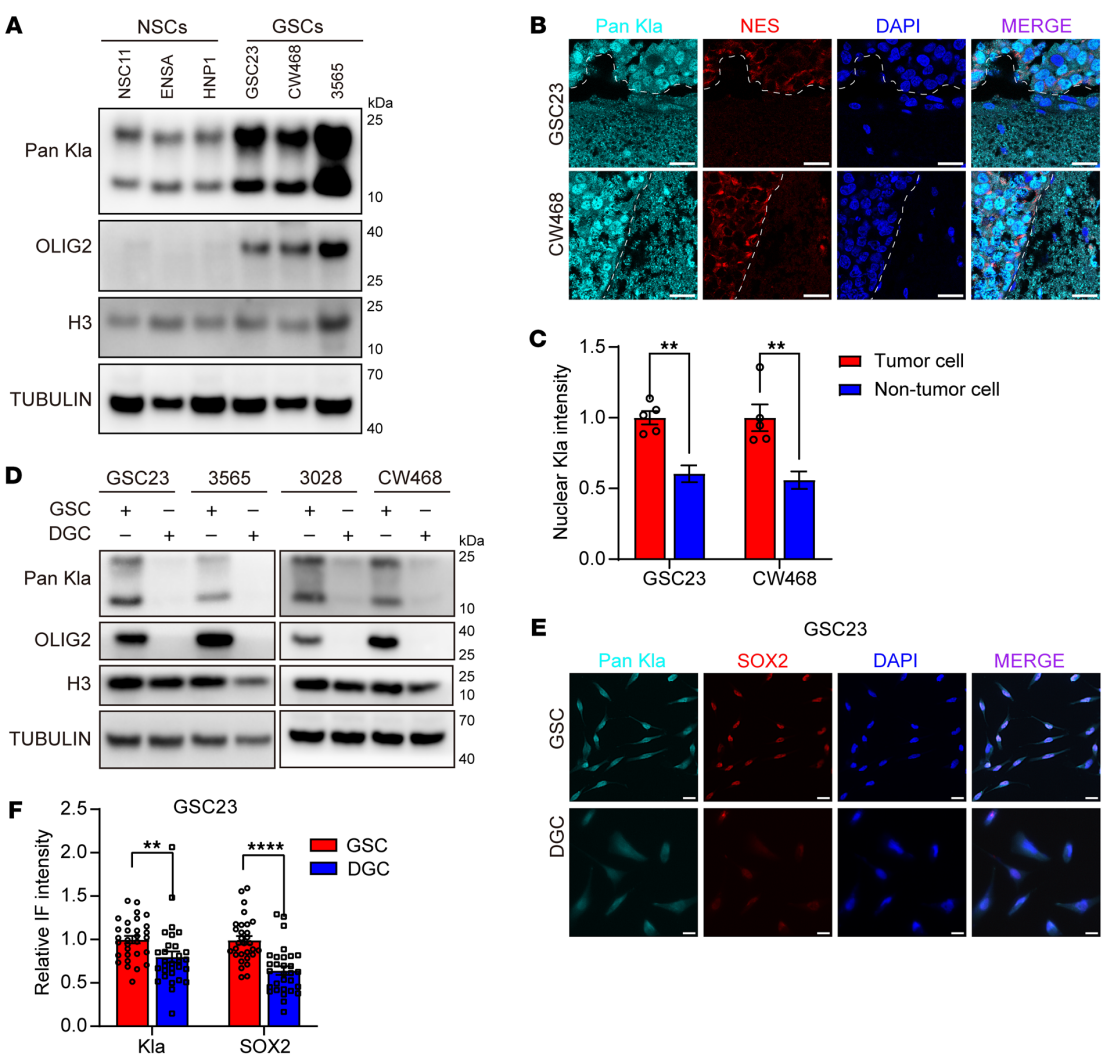
Histone lactylation levels are elevated in GBM cells. (**A**) Western blot of histone lactylation (Kla) and OLIG2 in 3 different NSCs (NSC11, ENSA, and hNP1) and 3 GSCs (GSC23, CW468, and 3565). Histone 3 (H3) and Tubulin were used as loading controls. (**B**) Immunofluorescence staining of protein lactylation (Pan Kla) in GSC23 and CW468 intracranial tumor xenografts. Human nestin (NES) marks tumor cells. DAPI marks nuclei. The brain-tumor border is demarcated by white dashed lines. Scale bars: 20 μm. (**C**) Graphic quantification of nuclear histone lactylation staining in **B** (*t* tests; *n* = 5/group). (**D**) Western blot of histone lactylation (Kla) and OLIG2 in 4 different GSCs (GSC23, 3565, 3028, and CW468) and paired DGCs. Histone 3 and Tubulin were used as loading controls. (**E**) Immunofluorescence staining of lysine lactylation (Kla) and SOX2 in GSC23 and paired DGC. DAPI was used to mark nuclei. Scale bars: 20 μm. (**F**) Statistical analysis of nuclear Kla levels in GSC23 and paired DGCs (*t* tests; *n* = 30/group). **P < 0.01; ****P < 0.0001.

Consistent with oncogenic metabolic reprogramming and Warburg metabolism, cultured GSCs had higher lactate levels than human neural stem cells (NSCs), the putative cell of origin for GBM (Supplemental Figure 1A; supplemental material available online with this article; https://doi.org/10.1172/JCI176851DS1). Elevated lactate production in tumor cells has been proposed to promote histone lactylation (21). Indeed, GSCs displayed increased histone lactylation relative to NSCs on immunoblot (Figure 1A). Immunofluorescence of GSC-derived xenografts showed increased histone lactylation in nuclei compared with normal brain cells in vivo (Figure 1, B and C). To determine whether histone lactylation was specific to GSCs and their epigenetic landscape (25), we induced GSC differentiation into differentiated GBM cells (DGCs). The effectiveness of differentiation was confirmed by downregulation of GSC markers OLIG2 and SOX2 in DGCs (Figure 1, D–F). GSCs had higher histone lactylation levels than DGCs (Figure 1, D–F).

**Figure 2:**
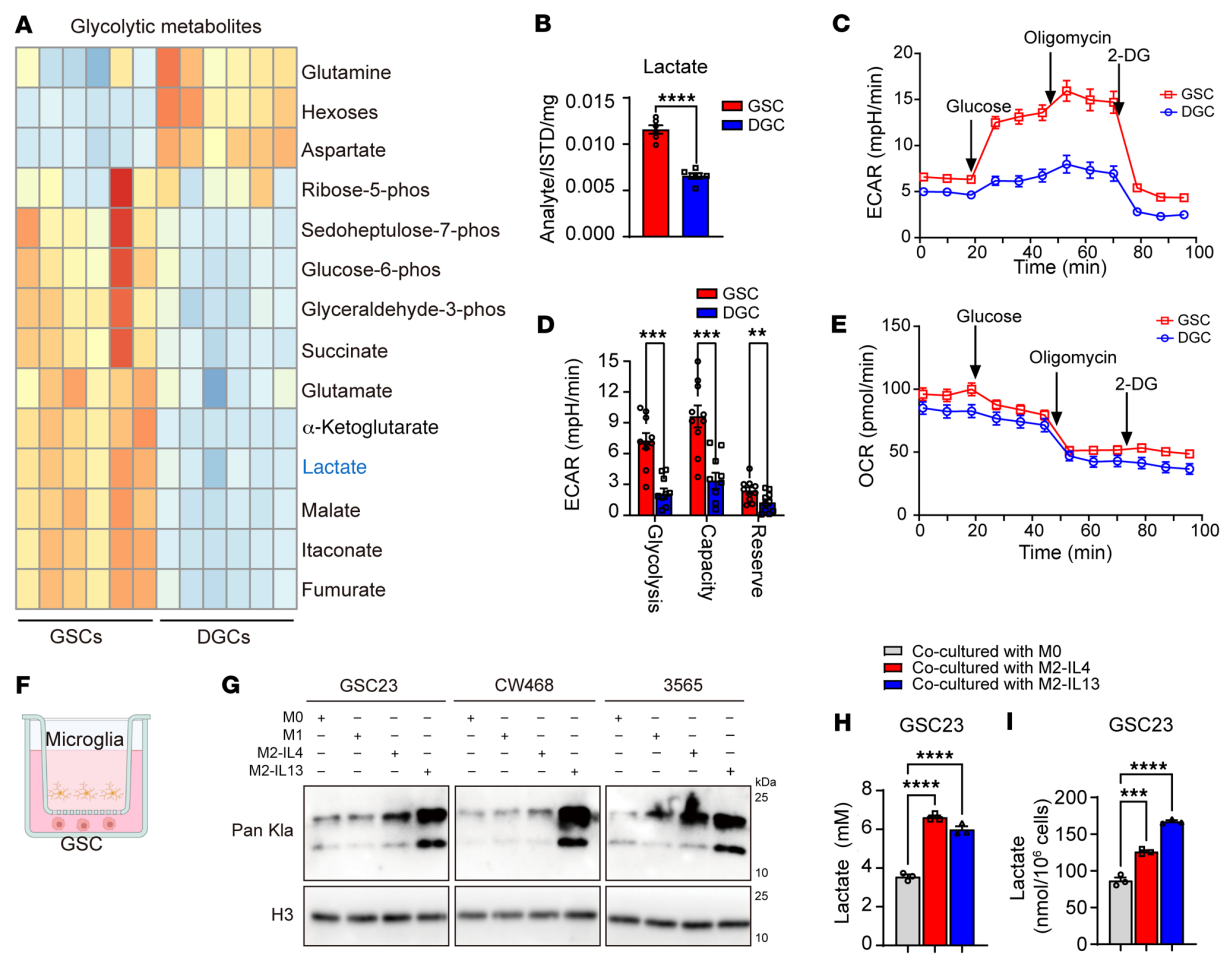
Elevated lactate promotes lactylation in GBM cells. (**A**) Heatmap shows MS analysis of glycolysis-related metabolites in GSC23 and DGC23. Red and blue designate higher and lower levels, respectively. (**B**) Graphic quantification of lactate levels in GSC23 and DGC23 (*t* test; *n* = 6/group). (**C**) ECAR values of matched GSC23 and DGC23 in Seahorse assays, after sequential injection of 20 mM glucose, 1 μM oligomycin, and 100 mM 2-deoxy-d-glucose (2-DG) (*n* = 10/group). (**D**) Quantification of glycolysis, glycolytic capacity, and glycolytic reserve in GSC23 and DGC23 in the Seahorse assay (*t* test; *n* = 10/group). (**E**) Oxygen consumption rate (OCR) values of matched GSC23 and DGC23 in Seahorse assays (*n* = 10/group). (**F**) Schematic showing the coculture of GSCs and microglia using Transwell inserts. (**G**) Western blot of histone lactylation in 3 GSCs (GSC23, CW468, and 3565) cocultured with M0 microglia (HMC3), M1-like microglia induced by LPS, or M2-like microglia induced by either IL-4 (10 ng/ml IL-4, designated as M2–IL-4) or IL-13 (10 ng/ml IL-13, designated as M2–IL-13). Histone 3 was used as loading control. (**H** and **I**) Quantification of lactate concentration in the culture medium (**H**) and intracellularly in GSCs (**I**). GSC23 tumor cells were cocultured with M0 microglia or microglia induced toward an M2-like state through either IL-4 (M2–IL-4) or IL-13 (M2–IL-13). Coculture with M2 microglia increased both extracellular (*n* = 3/group; 1-way ANOVA; F[2, 6] = 153.9) and intracellular (*n* = 3/group; 1-way ANOVA; F[2, 6] = 192.2) lactate. **P < 0.01; ***P < 0.001; ****P < 0.0001.

To explore the underlying mechanisms of elevated histone lactylation in GSCs, we measured levels of key metabolites in GSCs and DGCs, including products of glycolysis, the pentose phosphate pathway, and the tricarboxylic acid cycle, by liquid chromatography–mass spectrometry (LC-MS), which revealed upregulation of glycolytic metabolites, including lactate, in GSCs (Figure 2, A and B). Accordingly, mRNA levels of enzymes involved in aerobic glycolysis were elevated in GSCs relative to DGCs (Supplemental Figure 1B). To further explore the mechanism of elevated lactate levels in GSCs compared with DGCs, we performed glycolysis stress tests using the Seahorse extracellular flux analyzer to measure glycolysis, glycolytic capacity, and glycolytic reserve in intact GSCs and DGCs. We observed that the extracellular acidification rate (ECAR) increased in GSCs compared with DGCs (Figure 2C), reflecting higher levels of glycolysis, glycolytic capacity, and glycolytic reserve compared with DGCs (Figure 2D). In contrast, oxygen consumption rate, which primarily reflects mitochondrial respiration, was only slightly higher in GSCs than DGCs (Figure 2E). Collectively, these results suggest elevated lactate levels and histone lactylation in GSCs.

Lactate dehydrogenase A (LDHA) converts pyruvate to lactate, thereby promoting histone lactylation (21, 26). We therefore interrogated *LDHA* expression levels on publicly available datasets. *LDHA* was elevated in GBM compared with normal brain (Supplemental Figure 2A). GBM has been classified into transcriptional subtypes (27, 28), among which the mesenchymal subtype has been associated with regions of hypoxia and inflammation (29). Interrogating The Cancer Genome Atlas (TCGA) GBM dataset for transcriptional subtypes revealed higher *LDHA* expression in mesenchymal tumors compared with classical and proneural subtypes (Supplemental Figure 2B). *LDHA* expression correlates with tumor grade, as GBM exhibits *LDHA* higher levels compared with low-grade gliomas and portends a poor prognosis in GBM patients (Supplemental Figure 2, C–F). Collectively, these results suggest that increased production of lactate and thus histone lactylation correlates with aggressive behavior in GBM.

To assess the function of LDHA, we knocked it down in 4 patient-derived GSCs, with knockdown efficiency verified by immunoblot (Supplemental Figure 2G). Reduced LDHA expression inhibited GBM cell proliferation (Supplemental Figure 2, H–K). Direct effects of lactate on tumor cell growth were measured with the addition of exogenous sodium lactate (NaLac) to the culture medium, which promoted GSC growth in vitro and increased histone lactylation (Supplemental Figure 2, L–P), suggesting cell-autonomous induction of tumor growth. In contrast, exogenous lactate had no effect on in vitro NSC proliferation or viability (Supplemental Figure 2, Q and R). Collectively, these results suggest that lactate and histone lactylation promote tumor growth through direct effects on GSCs.

### Histone lactylation in GSCs inhibits phagocytosis by microglia and promotes in vivo tumor growth.

Monocyte-derived macrophages and microglia account for up to 50% of the cellular population in GBM and promote tumor growth (30). During development, microglia provide lactate to neurons as a carbon and energy source (31). Similarly, macrophages and microglia produce lactate in tumors (32). Therefore, we tested to determine whether microglia regulate histone lactylation levels in GSCs. Microglia and macrophages display striking differences in their gene expression and cellular interactions depending on their polarization (8). Therefore, we compared interactions between microglia cultured under conditions to generate M0, M1, and M2 states (33). The M1-like state was induced by LPS (34).The M2-like state was induced through conditioning with either IL-4 or IL-13 (33). GSC cells were then cultured in Transwell inserts at a 1:1 ratio with HMC3 microglial cells (Figure 2F), and GSCs were selectively harvested. M2-like microglia induced by either IL-4 or IL-13 increased histone lactylation levels in GBM cells relative to M0-like microglia (Figure 2G). To determine the source of lactate, we measured extracellular lactate concentrations in GSC23-microglia cocultures and intracellular lactate in GSCs. Induction of M2-like polarization by either IL-4 or IL-13 led to higher lactate levels in the culture medium and GSCs than M0-like microglia (Figure 2, H and I). Collectively, these data indicate that coculture of GSCs with immunosuppressive M2-like microglia increases their intracellular lactate levels and histone lactylation.

**Figure 3:**
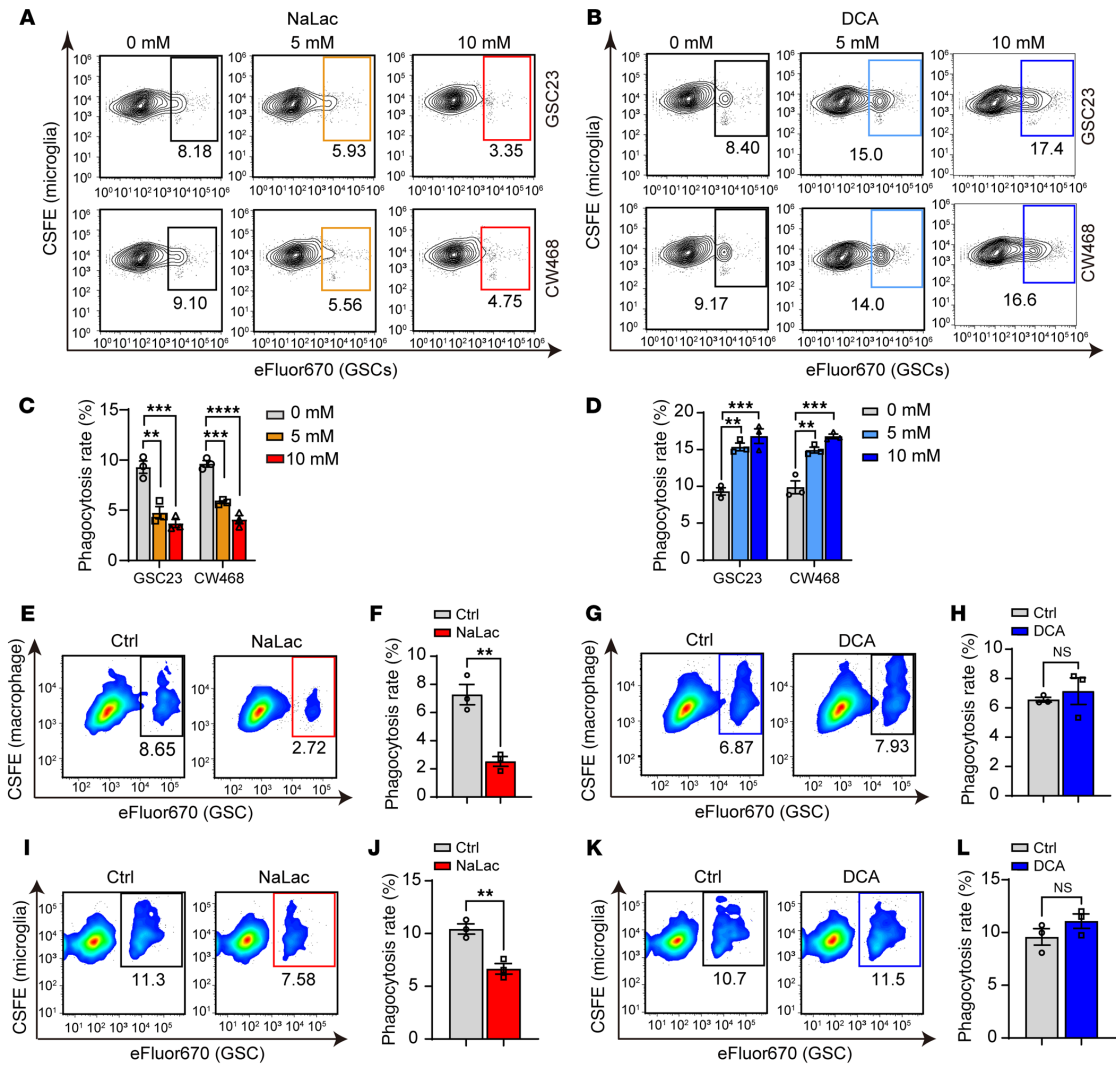
Histone lactylation regulates phagocytosis of GSCs by microglia in vitro and in vivo. (**A** and **B**) Representative flow cytometry plots of in vitro phagocytosis of GSCs (stained with eFluor670) by microglia (stained with carboxyfluorescein succinimidyl ester [CSFE]) after GSC pretreatment with NaLac (**A**) or DCA (**B**) for 24 hours. (**C** and **D**) Statistical analysis of phagocytosis assays of microglia against GSCs pretreated with NaLac (**C**) (*n* = 3/group; 1-way ANOVA; F[2, 6] = 29.39 for GSC23), (F[2, 6] = 84.10 for CW468) or DCA (**D**) (*n* = 3 /group; 1-way ANOVA; F[2, 6] = 31.63 for GSC23, F[2, 6] = 38.02 for CW468). (**E**) Representative flow cytometry plot of in vitro phagocytosis of GSC23 (stained with eFluor670) by macrophage (stained with CSFE). GSC23 were pretreated with 10 mM NaLac for 24 hours before the phagocytosis assay. (**F**) Graphic quantification of the assay in **E** (*n* = 3/group; *t* test). (**G**) Representative flow cytometry plot of macrophage (stained with CSFE) phagocytosis of eFluor670-stained GSC23 pretreated with 10 mM DCA for 24 hours. (**H**) Graphic quantification of the assay in **G** (*n* = 3/group; *t* test). (**I**) Representative flow cytometry plots of in vitro phagocytosis of GSC23 by microglia. Microglia were pretreated with vehicle (PBS) or 10 mM NaLac for 24 hours before coculture. GSCs and microglia were stained with eFluor670 and CFSE, respectively. (**J**) Graphic quantification of GSC23 phagocytosis by microglia in **I** (*n* = 3/group; *t* test). (**K**) Representative flow cytometry plots of microglial phagocytosis of GSCs. Microglia were pretreated with PBS or DCA for 24 hours before phagocytosis measurements. GSCs and microglia were stained with eFluor670 and CFSE, respectively. (**L**) Graphic quantification of GSC23 phagocytosis by microglia in **K** (*n* = 3/group; *t* test). **P < 0.01; ***P < 0.001; ****P < 0.0001.

The innate immune functions of macrophages and microglia contribute to tumor control in part through the phagocytosis of tumor cells. To investigate potential effects of lactate in this process, HMC3 microglia were fluorescently labeled with CFSE, then cocultured with 2 patient-derived GSCs that were labeled with eFluor670. Tumor cell phagocytosis was then quantified using flow cytometry to identify dually labeled cells. To test the effects of lactate on phagocytosis, we pretreated either cell type with exogenous lactate (NaLac) or DCA, which decreases glycolysis and lactate levels (35), independently before coculture. Treatment of GSCs (GSC23 and CW468) with NaLac for 24 hours inhibited their phagocytosis by microglia, while DCA produced the opposite effect (Figure 3, A–D). DCA also repressed histone lactylation in GSCs (Supplemental Figure 2S).

As monocyte-derived macrophages are dominant myeloid infiltrates in GBM, we also incorporated macrophages in our studies. THP-1 monocytes were differentiated into macrophages by 48-hour incubation with 200 nM phorbol 12-myristate 13-acetate, as previously described (36) (Supplemental Figure 2T). GSC23 were pretreated with either NaLac or DCA to increase or decrease lactate levels, respectively. Macrophages and GSCs were then cocultured and analyzed by flow cytometry to determine the rate of phagocytosis. Flow cytometry showed that NaLac reduced macrophage phagocytosis (Figure 3, E and F), but DCA did not influence phagocytosis by macrophages (Figure 3, G and H). Collectively, these findings suggest that lactate in the microenvironment and histone lactylation suppress phagocytosis of tumor cells, thereby contributing to immune evasion, in addition to tumor-cell– intrinsic tumorigenic effects.

Prior work showed that microglial histone lactylation induced by microenvironmental lactate promotes M2 polarization and reduces tumor phagocytosis (16). We therefore tested to determine whether lactylation changes in microglia can also regulate phagocytosis. Immunoblotting showed that treatment with 10 mM NaLac for 24 hours increased histone lactylation levels in human HMC3 microglia (Supplemental Figure 2U) and inhibited their in vitro phagocytosis of GSCs (Figure 3, I and J). Conversely, when we blocked lactate production in microglia by treatment with 10 mM DCA for 24 hours, histone lactylation decreased (Supplemental Figure 2V) without significant change in phagocytosis (Figure 3, K and L).

**Figure 4:**
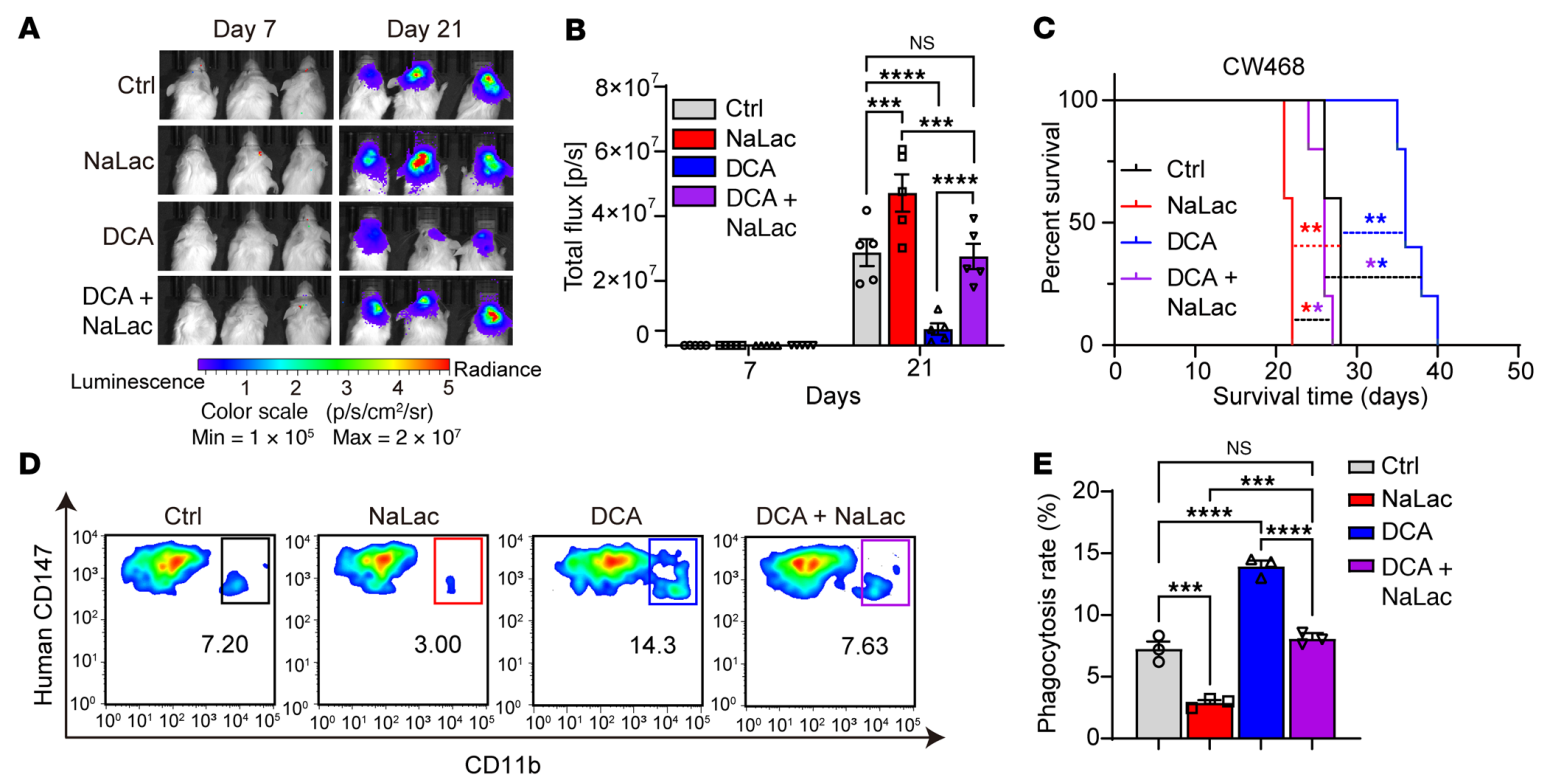
Histone lactylation regulates phagocytosis of GSCs by microglia and macrophages in vivo. (**A**) Representative bioluminescent images on days 7 and 21 of mice bearing tumors derived from CW468. Mice were treated with vehicle (PBS), NaLac (1 g/kg/d), DCA (150 mg/kg/d), or DCA (150 mg/kg/d) plus NaLac (1 g/kg/d) from day 7 until the experimental endpoint. (**B**) Quantification of bioluminescent signals in CW468 tumor-bearing mice at days 7 and 21 (*n* = 5/group; 2-way ANOVA, F[3, 32] = 17.19). (**C**) Kaplan-Meier survival curves of tumor-bearing mice implanted with CW468 cells treated with PBS vehicle, NaLac, DCA, or DCA plus NaLac from day 7 (*n* = 5/group; log-rank tests). (**D**) Representative flow cytometry plot of in vivo GSC phagocytosis by microglia in CW468 tumor-bearing mice treated with either vehicle (PBS), NaLac, DCA, or DCA plus NaLac. Tumor cells were identified with staining for human CD147. Murine microglia/macrophages were identified with CD11b. (**E**) Quantification of in vivo phagocytosis in CW468 tumor-bearing mice treated with either vehicle (PBS), NaLac, DCA, or DCA plus NaLac (*n* = 5/group; 1-way ANOVA; F[3, 8] = 114.4). **P < 0.01; ***P < 0.001; ****P < 0.0001.

To extend these findings in vivo, patient-derived GSCs were transduced with a luciferase reporter and then implanted into the brains of immunodeficient NSG mice. In these mice, phagocytosis of human cells by mouse macrophages and microglia is compromised due to a mutation in mouse SIRPα that allows recognition of human CD47, in addition to defective adaptive immunity due to loss of IL-2Rγ (37). However, innate immune function is partially intact in NSG mice. After permitting the development of GSC-derived tumors for 7 days, tumor-bearing mice were then randomized into 4 treatment groups: vehicle control, NaLac (1 g/kg/d), DCA (150 mg/kg/d), or combined NaLac (1 g/kg/d) and DCA (150 mg/kg/day), each administered daily by intraperitoneal injection. Concordant with in vitro effects, NaLac promoted, whereas DCA inhibited tumor growth in vivo measured by in vivo imaging system (IVIS) imaging (Figure 4, A and B). Differences detected in tumor volumes translated into survival differences; NaLac reduced the survival of tumor-bearing mice, whereas DCA prolonged it (Figure 4C). Given lactate and DCA have opposing effects on lactylation and tumor growth, combined treatment with NaLac and DCA caused essentially identical tumor growth and survival as the control arm (Figure 4, A–C).

To correlate these findings with histone lactylation, tumors from each group were harvested and histone lactylation quantified by immunofluorescence microscopy. NaLac increased nuclear histone lactylation of tumor cells, whereas DCA inhibited it (Supplemental Figure 3, A and B). Combined treatment with NaLac and DCA produced histone lactylation levels similar to those of control tumors (Supplemental Figure 3, A and B). Based on the effects of lactate on microglial/macrophage phagocytosis in vitro, we investigated the impact of lactate modulation on in vivo phagocytosis of tumor cells. Tumors harvested from each in vivo treatment group were subjected to dual flow cytometric analysis to identify tumor cells using a human-specific CD147 antibody and microglia/macrophages using a CD11b antibody. NaLac inhibited in vivo phagocytosis of GSCs, whereas DCA promoted it (Figure 4, D and E). Combined treatment with NaLac and DCA had effects similar to those of control conditions (Figure 4, D and E). Collectively, these data show that lactate and histone lactylation promote tumor growth through combined effects on GSC proliferation and GSC phagocytosis by microglia/macrophage in vitro and in vivo.

### Histone lactylation promotes immune-suppressive pathways in GBM.

**Figure 5:**
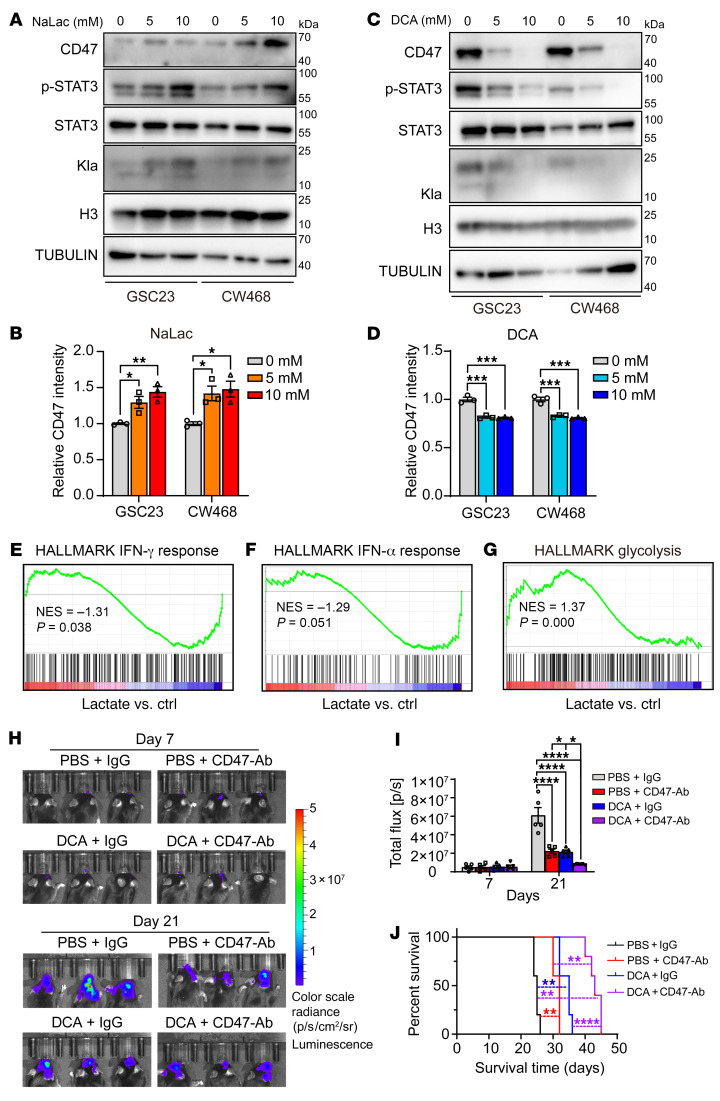
Histone lactylation regulates immune evasion pathways in GBM cells. (**A**) Western blot of CD47, phosphorylated STAT3 (p-STAT3), STAT3, and Kla protein levels in GSC23 and CW468 treated with different concentrations of NaLac. Histone 3 and Tubulin were used as loading controls. (**B**) Quantification of effect of NaLac on membrane CD47 expression by flow cytometry (representative data shown in Supplemental Figure 3C) (*n* = 3/group; 1-way ANOVA; F[2, 6] = 12.36 for GSC23), (F[2, 6] = 8.913 for CW468). (**C**) Western blot of CD47, p-STAT3, STAT3, and Kla protein levels in GSC23 and CW468 treated with different concentrations of DCA. Histone 3 and Tubulin were used as loading controls. (**D**) Quantification of effect of DCA on membrane CD47 expression by flow cytometry (representative data shown in Supplemental Figure 3D) (*n* = 3/group; 1-way ANOVA; F[2, 6] = 54.92 for GSC23), F[2, 6] = 53.89 for CW468). (**E**–**G**) GSEA analysis shows that lactate stimulation was negatively related to IFN-γ response, IFN-α response, and glycolysis. (**H**) Representative bioluminescent images on days 7 and 21 of immunocompetent mice implanted with CT2A murine glioma cells. Tumor-bearing mice were treated with either PBS plus IgG (100 μg/mouse), PBS plus anti-CD47-Ab (100 μg/mouse), DCA (150 mg/kg/d) plus IgG (100 μg/mouse), or DCA (150 mg/kg/d) plus anti-CD47-Ab (100 μg/mouse) on days 7 and 14. (**I**) Quantification of bioluminescent signals in CT2A tumor-bearing mice on days 7 and 21 (*n* = 5/group; 2-way ANOVA; F[3, 32] = 23.15). (**J**) Kaplan-Meier survival curves of tumor-bearing mice implanted with CT2A cells treated with vehicle control, vehicle control (PBS) plus IgG, PBS plus anti-CD47-Ab, DCA plus IgG, or DCA plus anti-CD47-Ab (*n* = 5/group; log-rank tests). *P < 0.05; **P < 0.01; ***P < 0.001; ****P < 0.0001.

Prominent among immune evasion pathways in GBM and other cancers, CD47 provides a “don’t eat me” signal by binding SIRPα expressed on macrophages and microglia (8). Additionally, signal transducer and activator of transcription 3 (STAT3) activation (phosphorylation at Tyr705) in GBM cells promotes immune evasion associated with reduced numbers of phagocytes, decreased phagocytosis, and inhibition of T cell proliferation (38). Therefore, we tested to determine whether the effects of lactate and histone lactylation were associated with alterations in CD47 expression and STAT3 function. NaLac treatment of GSCs induced concentration-dependent increases in CD47 expression and STAT3 activation by immunoblot (Figure 5A) and plasma membrane CD47 expression by flow cytometry (Figure 5B and Supplemental Figure 3C). In contrast, DCA had opposite effects (Figure 5, C and D, and Supplemental Figure 3D). To address the potential mediators of these effects, we performed RNA-Seq on 3 patient-derived GSCs after NaLac treatment. Gene set enrichment analysis (GSEA) revealed that IFN-γ and IFN-α responses were inhibited by NaLac, whereas glycolysis was activated (Figure 5, E–G). Thus, histone lactylation in GSCs may influence the immune microenvironment in GBM via transcriptional programs regulated by interferon (39).

Anti-CD47 therapies have entered clinical trials, including in neuro-oncology, although initial results have been mixed (40). Given the induction of CD47 by lactate and repression by DCA, we hypothesized that attenuating lactate production could improve the efficacy of anti-CD47 agents. Thus, we interrogated the combinatorial benefit of DCA and CD47 targeting in an immunocompetent syngeneic tumor model utilizing mouse CT2A glioma cell implantation in the mouse brain. The combination of DCA and neutralizing CD47 antibody produced a more pronounced impairment of tumor growth relative to either agent alone, which translated into further prolongation of survival of tumor-bearing mice (Figure 5, H–J). Analysis of tumors harvested from each treatment arm revealed that combined DCA and anti-CD47 therapy increased CD4^+^ and CD8^+^ T cell infiltration relative to either treatment alone (Supplemental Figure 3, E–H). Thus, lactate modulates both adaptive and innate immune responses by inducing CD47 expression and STAT3 activation.

### CBX3 augments lactylation activity of EP300.

**Figure 6:**
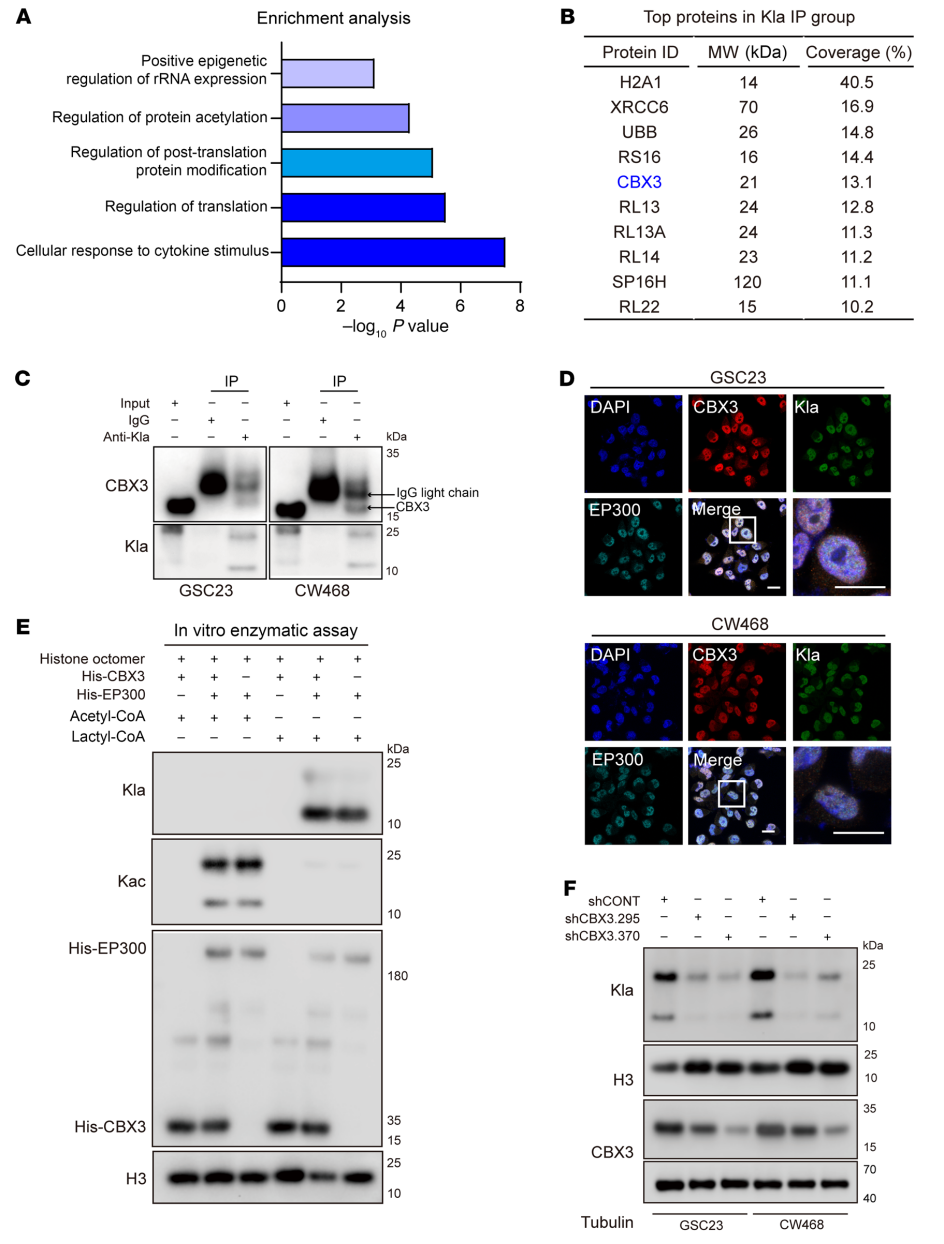
CBX3 promotes histone lactylation by increasing catalytic ability of EP300. (**A**) Enrichment analysis by Metascape shows gene ontology terms enriched among lactylated proteins. (**B**) Top 10 proteins IP with an antibody against lactylated lysine (Kla). (**C**) Co-IP analysis of Kla and CBX3 in GSC23 and CW468 cells with either an IgG control or anti-CBX3 antibody. (**D**) Immunofluorescence staining of CBX3, EP300, and Kla in GSC23 and CW468. DAPI marks nuclei. Scale bars: 10 μm. (**E**) Western blot shows that His-CBX3 protein increases the lactyl-transferase of EP300. CBX3 has no effect on the acetyl-transferase function of EP300. (**F**) Western blot shows the levels of histone lactylation in GSC23 and CW468 cells transduced with either shCONT, shCBX3.295, or shCBX3.370. Histone 3 and Tubulin were used as loading controls.

We next sought to determine molecular mechanisms regulating the tumorigenic effects of histone lactylation in GBM. To identify interactors with lactylated histone, we immunoprecipitated (IP) lactylated proteins from whole cell lysates by using an anti-lactylated lysine (Kla) antibody followed by MS (Supplemental Figure 4A). Quantitative MS analysis showed that 47 proteins were exclusively identified in the Kla IP group compared with IgG control (Supplemental Data Set 1). Pathway enrichment by Metascape (36) demonstrated enrichment of proteins related to regulation of ribosomal RNA (rRNA) expression, protein acetylation, posttranslational protein modification, translation, and response to cytokines in the Kla IP group (Figure 6A). The top 10 proteins were ranked by peptide percentage coverage (Figure 6B) and included chromobox protein homolog 3 (CBX3), which is a member of the heterochromatin-associated protein 1 (HP1) family that classically represses gene expression (41). The top hit was histone 2A type 1 (H2A1), which is 1 of the 5 main histone proteins involved in chromatin structure (42) and a lactylation target in macrophage (21), thus validating our MS findings. Other top targets have roles in DNA damage (XRCC6), proteasomal degradation (ubiquitin [UBB]), and protein translation (ribosomal protein RS16). While each of these targets may provide important directions in future studies, we focused on CBX3, also known as HECH or HP1γ, which binds DNA as a component of heterochromatin and is also connected to metabolism (43, 44). Aberrant CBX3 expression has been associated with tumor progression in several cancer types, including glioma and gastric cancer (45, 46). In contrast to its classical role as a transcriptional repressor, CBX3 can also activate gene expression (47). Given its role in regulating chromatin structure and binding with histones, we prioritized CBX3 for further analysis. CBX3 was co-IPed with anti-Kla antibody from 2 patient-derived GSCs (Figure 6C). The binding was confirmed by reverse IP for CBX3 (Supplemental Figure 4B). CBX3 also bound to SOX2 (Supplemental Figure 4B), a transcription factor expressed in GSCs (48). Thus, CBX3 may function concordantly with SOX2 to regulate histone lactylation and transactivation of SOX2 targets in GSCs.

CBX3 is a small protein without a catalytic domain for histone lactylation, so we hypothesized that CBX3 regulates histone lactylation indirectly. The HAT EP300 has been proposed as the enzyme that catalyzes histone lactylation (21), so we investigated potential interactions between CBX3 and EP300. Co-IP assays in GSCs transduced with Flag-tagged CBX3 (Flag-CBX3) demonstrated that CBX3 bound to endogenous EP300 (Supplemental Figure 4C). Similarly, hemagglutinin-tagged EP300 (HA-EP300) bound to FLAG-CBX3 in HEK293T cells (Supplemental Figure 4D). Immunofluorescence confocal microscopy in 2 GSCs demonstrated that CBX3, EP300, and lactylated histones colocalized in the nucleus (Figure 6D). Thus, CBX3 interacts with lactylated histones and the histone lactylation writer EP300.

EP300 catalyzes multiple acylation reactions, including acetylation, lactylation, propionylation, and butyrylation (49). Given the connection among CBX3, EP300, and lactylation, we hypothesized that CBX3 might increase the selective catalytic activity of EP300 in lactylation. Using in vitro enzymatic assays, we found that CBX3 selectively increased histone lactylation, but not acetylation by EP300 (Figure 6E). CBX3 knockdown in GBM cells inhibited histone lactylation (Figure 6F), which was confirmed by immunofluorescence staining of histone lactylation in the nucleus (Supplemental Figure 4, E–H). In reciprocal gain-of-function studies, CBX3 overexpression increased histone lactylation in GBM cells (Supplemental Figure 4I). Thus, CBX3 promotes histone lactylation through induction of EP300 substrate selectivity toward lactyl-CoA.

### CBX3 promotes tumor growth and phagocytosis by regulating histone lactylation.

Given the connection among histone lactylation, microglial/macrophage phagocytosis, and CBX3, we tested to determine whether CBX3 regulates phagocytosis of tumor cells. *CBX3* was elevated in GBM compared with normal brain tissues in TCGA, regardless of molecular subtype (Supplemental Figure 5, A and B). *CBX3* positively correlated with glioma tumor grade (Supplemental Figure 5, C and D). On single-cell RNA-Seq analysis of GBM tumors (GSE84465), *CBX3* was expressed relatively more in neoplastic cells relative to nonmalignant cells (Supplemental Figure 5E). Single-cell analysis from another independent dataset (50) suggested that GSCs highly express *CBX3* compared with non-GSCs (Supplemental Figure 5F). In both the TCGA and Rembrandt databases (51), *CBX3* expression portended poor prognosis for GBM patients (Supplemental Figure 5, G and H). In loss-of-function studies, CBX3 knockdown inhibited GSC growth and sphere formation in vitro (Supplemental Figure 5, I–L), suggesting that CBX3 has tumor cell-autonomous tumorigenic actions.

**Figure 7:**
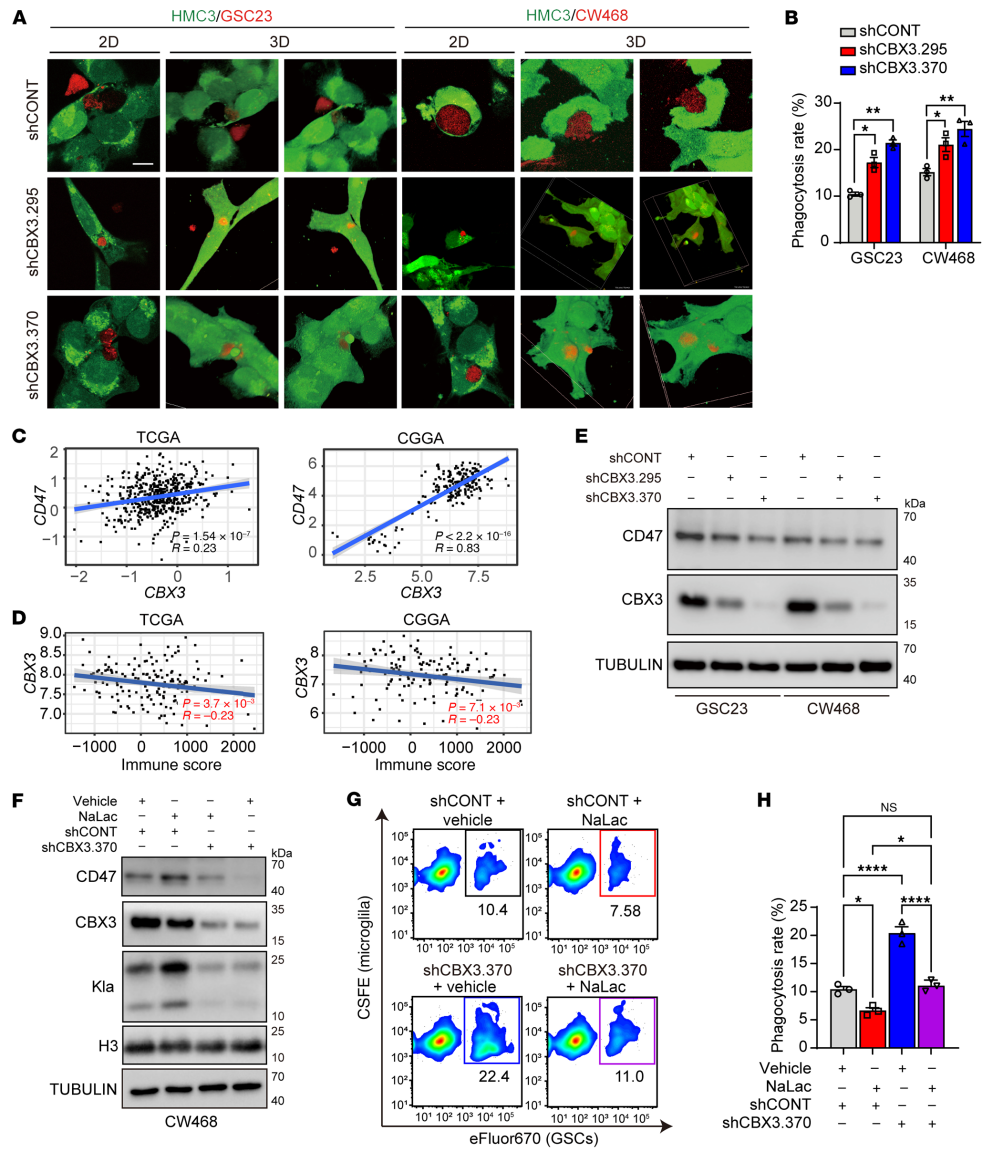
CBX3 knockdown in GBM cells regulates their phagocytosis by microglia. (**A**) 2D confocal microscopy images and their 3D reconstructions demonstrate effects of either shCONT or shCBX3 on phagocytosis of eFluor670-stained GBM cells (red) by CSFE-stained HMC3 microglia (green) in vitro. (**B**) Quantification of GBM cell phagocytosis by microglia in **A** (*n* = 3/group; 1-way ANOVA; F[2, 6] = 51.45 for GSC23, F[2, 6] = 12.17 for CW468). (**C**) Correlation between *CD47* and *CBX3* expression in TCGA and Chinese Glioma Genome Atlas (CGGA) databases. Data (normalized count value) were downloaded from GlioVis. (**D**) Correlation between immune score and *CBX3* expression in TCGA and CGGA databases. (**E**) Western blots of the protein levels of CBX3 and CD47 in GSC23 and CW468 cells transduced with either shCONT or shCBX3. Tubulin was used as loading control. (**F**) Representative Western blot of protein levels of CD47, CBX3, and histone lactylation in CW468 transduced with either a control shRNA sequence (shCONT) or shCBX3.370, then treated with either vehicle control or 10 mM NaLac for 24 hours. Histone 3 and Tubulin were used as loading controls. (**G**) Representative flow cytometry plots of eFluor670-stained CW468 cell phagocytosis by microglia (HMC3) stained with CSFE. Cell treatment conditions are the same as in **F**. (**H**) Quantification of relative phagocytosis rates in **G** (*n* = 3/group; 1-way ANOVA; F[3, 8] = 65.12). *P < 0.05; **P < 0.01; ****P < 0.0001.

Since histone lactylation promotes tumor growth via both tumor cell-autonomous and immunomodulatory mechanisms, we next investigated the functional role of CBX3 in tumor cell phagocytosis. When we cocultured microglia and patient-derived GSCs at a ratio of 1:1 (50,000 cells/well each), CBX3 knockdown in GSCs increased their microglial engulfment, as assayed with confocal microscopy (Figure 7, A and B) and flow cytometry (Supplemental Figure 6, A and B). Given the role of CD47 in phagocytosis, we then tested to determine whether CBX3 regulates CD47 expression. *CBX3* and *CD47* mRNA positively correlated in GBM datasets (Figure 7C), while *CBX3* negatively correlated with an immune score (52) that represents immune cell infiltration in GBM tumor tissue (Figure 7D). CBX3 knockdown in GSCs decreased CD47 expression in immunoblot and flow cytometry assays (Figure 7E and Supplemental Figure 6, C and D). We then tested to determine whether CBX3 knockdown interfered with NaLac-induced upregulation of CD47. Indeed, loss of CBX3 prevented the increase in histone lactylation and CD47 expression (Figure 7F) and lessened the decrease in phagocytosis (Figure 7, G and H) brought about by NaLac. Thus, CBX3 regulates histone lactylation to contribute to tumor growth through both tumor cell-autonomous and phagocytosis evasion mechanisms.

**Figure 8:**
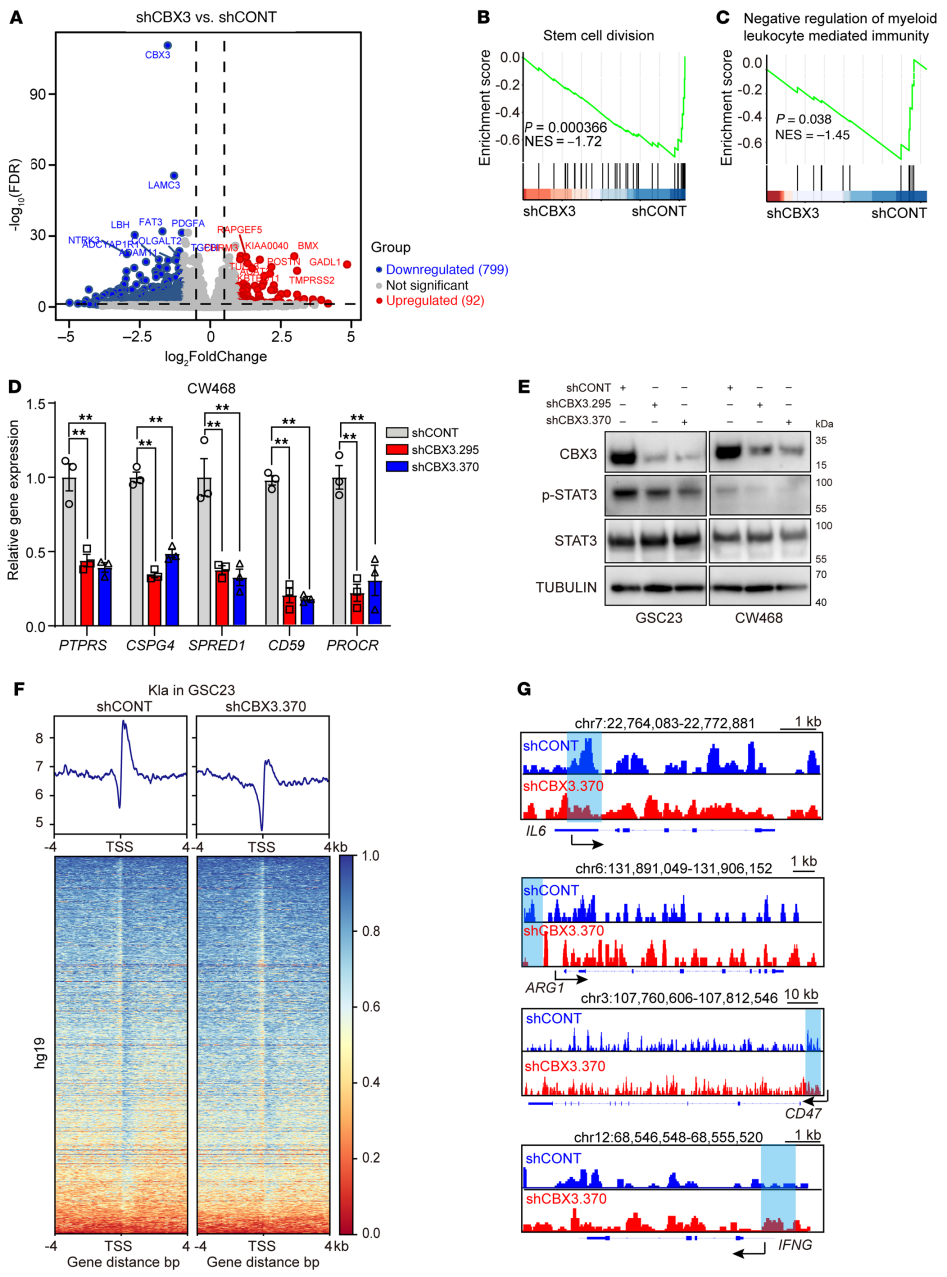
*CBX3* regulates immune-regulated pathways by histone lactylation. (**A**) Volcano plot shows the differential gene expression in GSC23 and CW468 cells transduced with either shCONT or shCBX3. The top 10 downregulated and upregulated genes ranked by *P* value are labeled. (**B** and **C**) GSEA analysis shows that CBX3 knockdown was negatively related to stem cell division and negative regulation of myeloid leukocyte–mediated immunity. (**D**) qRT-PCR analysis of *PTPRS*, *CSPG4*, *SPRED1*, *CD59* and *PROCR* in CW468 transduced with either shCONT or shCBX3. *ACTB* (encoding β-actin) was used as internal control (*n* = 3/group; 1-way ANOVAs; F[2, 6] = 31.05 for *PTPRS*, F[2, 6] = 152.1 for *CSPG4*, F[2, 6] = 21.88 for *SPRED1*, F[2, 6] = 165.7 for *CD59*), F[2, 6] = 27.60 for *PROCR*). (**E**) Western blot shows the protein levels of CBX3, p-STAT3, and STAT3 in GSC23 and CW468 cells transduced with either shCONT or shCBX3. Tubulin was used as loading control. (**F**) ChIP-Seq density heatmaps in GSC23 transduced with either shCONT or shCBX3, ranked by Kla read intensity, within ±4 kb of TSSs. (**G**) ChIP-Seq tracks showing Kla peaks in the promoter regions of *IL6*, *ARG1*, *CD47*, and *IFNG*.

To investigate the molecular mechanisms downstream of CBX3, we performed RNA-Seq of GSCs upon CBX3 knockdown. Differentially regulated genes (DEGs) were mapped on a volcano plot, which revealed more genes were downregulated than upregulated after CBX3 knockdown (Figure 8A), consistent with histone lactylation being an activating histone modification (21). GSEA analysis revealed changes related to stem cell division and regulation of myeloid leukocyte–mediated immunity (Figure 8, B and C). CBX3 knockdown decreased expression of protumorigenic interleukins (*IL4*, *IL10*, and *IL13*) and increased *IFNG* expression, as measured by quantitative reverse-transcriptase PCR (qRT-PCR) (Supplemental Figure 6, E and F). Other regulators of tumor biology, including *PTPRS*, *CSPG4*, *SPRED1*, *CD59*, and *PROCR*, were all downregulated upon CBX3 knockdown (Figure 8D). GSEA revealed that CBX3 knockdown modulated expression of genes related to tumor escape from immune attack, KRAS signaling, IL-6 receptor-ligand interaction, and STAT3 activity (Supplemental Figure 6, G–J). CBX3 knockdown inhibited STAT3 phosphorylation in GSCs (Figure 8E). To determine how CBX3 regulates gene expression, we mapped genome-wide histone lactylation after CBX3 knockdown using ChIP-Seq in GSCs. CBX3 knockdown resulted in prominent loss of lactylation at transcriptional start sites (TSSs) (Figure 8F). Granular analysis of Kla peaks upon CBX3 knockdown showed decreases near the promoter regions of *IL6*, *ARG1*, and *CD47*, whereas peaks near the promoter region of *IFNG* increased (Figure 8G). These findings suggest CBX3 regulates histone lactylation to fine-tune expression of genes related to immune function.

### CBX3 knockdown increases phagocytosis in vivo and prolongs survival of tumor-bearing mice.

**Figure 9:**
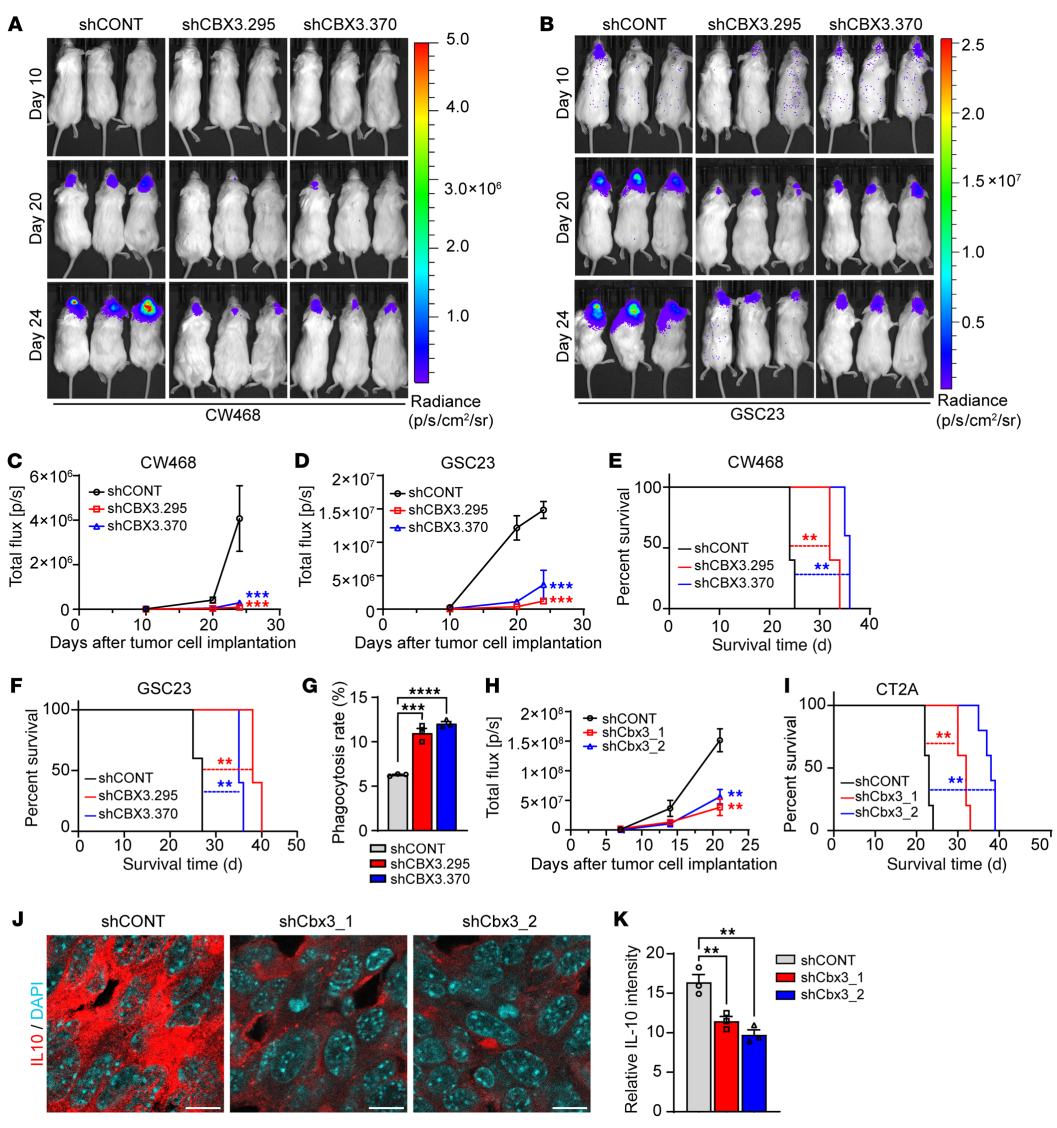
CBX3 knockdown increases phagocytosis in vivo and prolongs survival of tumor-bearing mice. (**A** and **B**) Representative bioluminescent images of mice intracranially implanted with CW468 (**A**) or GSC23 (**B**) cells transduced with either shCONT, shCBX3.295, or shCBX3.370 lentiviruses, on days 10, 20, and 24 after tumor cell implantation. (**C** and **D**) Quantification of bioluminescent signals in CW468 (**C**) or GSC23 (**D**) tumor-bearing mice at days 10, 20, and 24 (*n* = 5/group; 2-way ANOVA; F[4, 24] = 6.854 in **C**, F[4, 24] = 16.03 in **D**). (**E** and **F**) Kaplan-Meier survival curves of tumor-bearing mice implanted with CW468 (**E**) or GSC23 (**F**) cells transduced with either shCONT, shCBX3.295, or shCBX3.370 viruses (*n* = 5/group; log-rank tests). (**G**) Quantification of flow cytometric analysis of in vivo phagocytosis of CD147-positive GSCs by CD11b-positive microglia in NSG tumor-bearing mice (representative data shown in Supplemental Figure 7C) (*n* = 3/group; 1-way ANOVA; F[2, 6] = 83.44). (**H**) Quantification analysis of bioluminescent signals in CT2A tumor-bearing mice on days 7, 14, and 21 after tumor cell implantation (*n* = 5/group; 2-way ANOVA; F[4, 24] = 9.116). (**I**) Kaplan-Meier survival curves of tumor-bearing mice implanted with CT2A cells transduced with control virus or murine Cbx3 knockdown (shCBX3_1 or shCbx3_2) virus (*n* = 5/group; log-rank tests). (**J**) Representative immunofluorescence staining images for IL-10 in CT2A allografts transduced with either control shCONT or 1 of 2 nonoverlapping shRNAs targeting mouse Cbx3 (shCBX3_1 or shCbx3_2). DAPI marks nuclei. Scale bars: 10 μm. (**K**) Graphic quantification of IL-10 immunofluorescence intensity (*n* = 3/group; 1-way ANOVA; F[2, 6] = 20.71). **P < 0.01; ***P < 0.001; ****P < 0.0001.

We next investigated the effects of CBX3 on tumor growth in vivo. CBX3 knockdown in CW468 and GSC23 cells suppressed in vivo tumor growth (Figure 9, A–D), which translated into increased survival of tumor-bearing mice (Figure 9, E and F). Loss of CBX3 was associated with decreased histone lactylation (Supplemental Figure 7, A and B) and increased the prevalence of tumor cells engulfed by microglia/macrophages in vivo (Figure 9G and Supplemental Figure 7C).

Next, we explored the role of CBX3 in vivo with an intracranial murine glioma line (CT2A) grown in a syngeneic host. Efficiency of mouse Cbx3 knockdown was confirmed by both mRNA (qRT-PCR) and protein (immunoblot) analysis (Supplemental Figure 7, D and E). Cbx3 knockdown inhibited in vivo tumor growth (Figure 9H and Supplemental Figure 7F) and increased survival of tumor-bearing mice (Figure 9I). To determine whether knockdown of Cbx3 in murine glioma cell lines regulates phagocytosis, we collected the brains from mice bearing luciferase-expressing CT2A tumors and assayed phagocytosis with flow cytometry, using anti-luciferase antibody to detect tumor cells and antibodies against microglial markers CD11b and Tmem119, showing that knockdown of murine Cbx3 in CT2A cells increased phagocytosis (Supplemental Figure 7, G–J). To explore the mechanism underlying increased phagocytosis upon Cbx3 knockdown in CT2A cells, we measured MHC II expression in microglia, in which inflammation is known to upregulate MHC II (53). Cbx3 knockdown in CT2A cells increased in vivo MHC II expression in microglia (Supplemental Figure 8, A and B).

Concordant with the effects of Cbx3 on antitumor immune responses, tumors harvested from Cbx3-knockdown tumors displayed increased infiltration of CD4^+^ and CD8^+^ lymphocytes compared with control shRNA, as assayed with immunofluorescence microscopy (Supplemental Figure 8, C and D) and flow cytometry (Supplemental Figure 8, E and F). Infiltration was associated with increased IFN-γ expression in T cells (Supplemental Figure 8, G and H), suggesting augmentation of antitumor effects of T cells (54). Cbx3 knockdown in CT2A-derived tumors also reduced levels of the immunosuppressive cytokine IL-10 (Figure 9, J and K). These results suggest that CBX3 promotes tumor growth and immune evasion in vivo.

**Figure 10:**
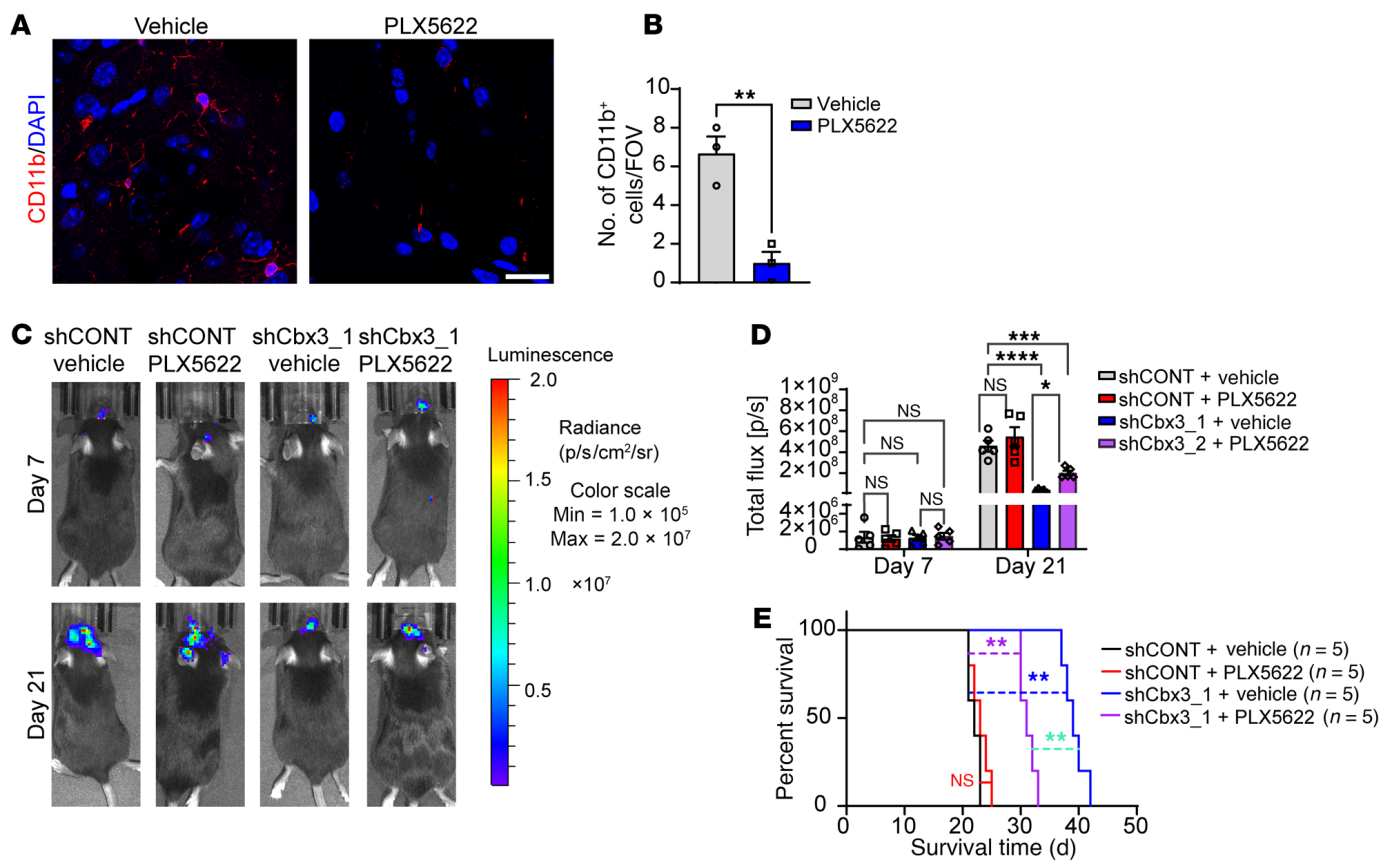
Depletion of microglia by PLX5622 compromises the inhibitory effects of Cbx3 knockdown on tumor growth in vivo. (**A**) Immunofluorescence imaging of CD11b microglia in murine brains following treatment with vehicle (sterilized water) or PLX5622 (50 μg/g daily) for 2 weeks. DAPI marks nuclei. Scale bar: 20 μm. (**B**) Statistical analysis of number of CD11b-positive cells (no.) per field of view (FOV) in mice treated with PLX5622 or control vehicle (*n* = 3/group; *t* test). (**C**) Representative in vivo bioluminescence images of CT2A intracranial tumors in C57BL6J mice pretreated with either vehicle or PLX5622. Images were obtained on days 7 and 21 after tumor cell implantation. CT2A cells were transduced with shCONT or shCbx3_1 before implantation. (**D**) Graphic quantification of bioluminescent signals of CT2A tumors transduced with shCONT or shCbx3_1 lentivirus. Mice were pretreated with vehicle (sterilized water) or PLX5622 (50 μg/g daily) for 2 weeks prior to intracranial tumor implantation (*n* = 5/group; 2-way ANOVAs; F(3, 16) = 18.36 on day 21). (**E**) Kaplan-Meier survival curves of C57BL6J mice bearing CT2A intracranial allografts. Mice were pretreated with either vehicle or PLX5622 for 2 weeks prior to tumor implantation. CT2A cells were transduced with either shCONT or shCbx3_1 (*n* = 5/group; log-rank tests). *P < 0.05; **P < 0.01; ***P < 0.001; ****P < 0.0001.

To determine the role of microglia in mediating the effects of CBX3, we performed pharmacological depletion of microglia in vivo using PLX5622 by intraperitoneal injection, as previously described (55, 56). To confirm successful depletion of microglia, we sacrificed a subset of mice in vehicle control and PLX5622 treatment groups prior to tumor implantation and performed immunofluorescence staining for microglia (Figure 10, A and B). The remaining mice were randomized to receive CT2A murine glioma cells transduced with either an shRNA control (shCONT) or Cbx3-targeting shRNA (shCbx3_1). Microglia depletion diminished the effects of Cbx3 knockdown on tumor growth (Figure 10, C and D) and survival (Figure 10E), a finding consistent with dual tumor cell-autonomous and immune-mediated actions of CBX3.

## Discussion

Tumor metabolism has been considered a downstream manifestation of oncogenic pathways, but increasing evidence supports metabolic dysregulation as a driving force in tumor initiation and progression. Oncometabolites generated through dysregulated metabolism may serve as cofactors for chromatin regulators, leading to alterations in cell state. For example, IDH mutations generate 2-hydroxyglutarate, which reprograms the epigenome toward tumorigenesis in leukemia and glioma (57, 58). In GBM, GSCs preferentially uptake lysine that undergoes degradation and induces accumulation of crotonyl-CoA, which represses the expression of endogenous retroviral elements to suppress antitumor responses (59). Here, we explore the role of lactate, a metabolite associated with aggressive tumor growth and immunosuppression in GBM (60). The view of lactate as an inert end product of glycolysis has been rapidly evolving to a more complex role in cellular metabolism and regulation of cell state. In GBM, lactate can rescue patient-derived xenograft cells from nutrient deprivation, in part via its utilization in the glycolytic pathway (15). Generation of lactate in glioma also stimulates accumulation of acetyl-CoA and an increase in histone acetylation, an activating chromatin modification (15). Thus, lactate can induce epigenetic changes to regulate complex gene-expression programs altering GSC biology, but also the interface between tumor cells and the tumor microenvironment.

Here, we add an additional dimension to the role of lactate in GBM biology. Lactate generated by tumor cells and microglia/macrophages induces histone lactylation in GSCs to activate expression of genes that generate an immunosuppressive interface between tumor and immune cells, including microglia/macrophages and T cells. Histone lactylation is upregulated in multiple cancers, including non–small cell lung cancer (61), liver cancer (62), ocular melanoma (24), and colon cancer (22). Lactate-derived histone lactylation was initially linked to induction of homeostatic genes involved in the late phase of M1 macrophage polarization (21). Monocyte-derived macrophages also generate lactate to promote immune cell–intrinsic lactylation associated with an immunosuppressive state (63). Our study extends the concept of lactylation as an epigenetic modifier beyond immune lineages to tumor cells. We find that glycolysis promotes elevated histone lactylation levels not only in immune cells, as previously reported (64, 65), but also in tumor cells. Mechanistically, our studies reveal a previously unreported role for CBX3 interacting with EP300, the histone acyltransferase (HAT) that acylates histones. In this interaction, CBX3, also known as HP1γ, biases EP300 to utilize lactyl-CoA rather than acetyl-CoA as substrate, thereby promoting epigenetic reprogramming through histone lactylation.

CBX3 is a multifunctional protein that participates in many cellular processes, including cell senescence, heterochromatin formation, DNA damage repair, and centrosome stability (66–70). CBX3 has been suggested to increase glioma growth by repressing transcription of E3 ubiquitin ligases that target epidermal growth factor receptor (EGFR) (71). Although CBX3 does not contain catalytic domains that mediate lactyltransferase activity (72), our results expand the function of CBX3 to include binding to EP300, which catalyzes multiple histone acylation reactions, including acetylation, lactylation, propionylation, and butyrylation (21, 73–75). While the mechanism by which CBX3 directs the specificity of EP300 toward lactylation remains to be elucidated, our findings suggest that CBX3 facilitates the impact of elevated lactate in regulating chromatin. This suggests that understanding the function of histone marks, like lactylation, will require more than simply modeling the direct cohort of writers, erasers, and readers of histone lactylation. Other roles of CBX3, such its association with heterochromatin, suggest that the histone regulation by CBX3 is integrated into the structural dynamics of the nucleus and may participate in the dynamic reconfiguration of euchromatic and heterochromatic states. Our demonstration of a role for CBX3 in glioma biology through actions on both proliferation of tumor cells and their interface with immune cells in the tumor microenvironment will enable future mechanistic studies that will dissect the impact of chromatin dynamics on complex cellular behaviors and interactions.

Although GBM was among the first solid tumors to undergo comprehensive molecular analysis, targeted therapies have offered limited benefit in clinical trials, prompting the development of other therapies, including immunotherapies. Unfortunately, oncoimmunology approaches have lacked consistent efficacy for GBM, suggesting that combinatorial approaches may improve the efficacy of immunotherapies. Here, we find that lactate generated by both tumor and immune cells in the microenvironment increases the expression of CD47 and activation of STAT3 in association with reduced microglial/macrophage phagocytosis of tumor cells. In an orthogonal observation, targeting lactate generation through pharmacologic inhibition of LDHA reduces chemokine secretion and recruitment of macrophage into tumors (76). Based on this background, we performed proof-of-principle studies that revealed that targeting lactate generation in combination with anti-CD47 therapy augments antitumor immune responses and inhibits tumor growth. Moving forward, this strategy can be used with pharmacological agents that target lactate production, including LDH inhibitors, which have entered preclinical and clinical development. We are heartened that DCA has been used in early clinical GBM studies with acceptable toxicity (14). The best-known limitation to DCA administration, observed both in preclinical and in clinical studies, is peripheral neuropathy due to sustained oxidative phosphorylation in cells producing ATP (77, 78). In this setting, contemporary administration of antioxidants represents a strategy to minimize the side effects of DCA-induced oxidative stress (78). As an example, glutathione transferase ζ 1 (GSTZ1), the first enzyme responsible for DCA clearance, may overcome DCA-induced oxidative damage. Indeed, a nonsynonymous functional SNP in human GSTZ1 has been reported to be associated with the clearance of DCA. Therefore, personalized DCA dosing, not only based on body weight, but also including GSTZ1 expression and activity, may minimize the dosage and side effects of DCA (79).

### Limitations of the current study.

The current study focuses on the interplay between tumor cells and microglia/macrophages, but lactate is also generated by other cell types in the brain (e.g., astrocytes) that serve critical roles in brain tumor biology. These cell types may also modulate tumor cell histone lactylation, and in turn, lactylated histones may regulate gene-expression programs that define tumor cell interactions with these components of the tumor microenvironment. This concept parallels metabolic regulation in normal brain development, where metabolic demands of specific cell types are met through both cell-autonomous activity and the interface with neighboring cells. For example, spatiotemporal partitioning of glycolytic and oxidative metabolism between astrocytes and neurons has been proposed through an astrocyte-neuron lactate shuttle (80). This interplay has also been proposed in gliomas, as monocarboxylate transporters (MCTs), specifically MCT1 and MCT4, are highly expressed in brain tumors and mediate proton-coupled transport of L-lactate, ketone bodies, and pyruvate (81). Future studies should include the study of lactate and histone lactylation in the context of resident glial lineages that contribute to the microenvironment in GBM.

The cohort of lactylated proteins we IP included H2A1, XRCC6, UBB, and RS16. XRCC6 binds to DNA during repair of DNA damage by forming heterodimers with XRCC5 (82). Ubiquitin B (UBB) plays a universal role in targeting cellular proteins for degradation by the 26S proteasome. Histones are known to undergo ubiquitin modifications (83). RS16 is a protein constituent of the 40S subunit of ribosomes (84). We therefore anticipate that future studies will include consideration of the role of lactylation of nonhistone proteins that regulate DNA damage responses, the proteasome, and ribosomes. Thus, we believe that our current observations are the beginning of a complex and rich connection between tumor metabolism and tumor biology.

We were excited to find that DCA and anti-CD47 antibodies demonstrated combinatorial benefit, but we recognize the limitations of this combination. Both DCA and agents targeting CD47 have dose-limiting toxicities. DCA induces peripheral neuropathy in preclinical and clinical studies (78). DCA-induced damage may be due to sustained oxidative phosphorylation in neural tissues (77). CD47 antibody therapy has been associated with hematotoxicity, particularly anemia, due to expression of CD47 on red blood cells (85). These toxicities are nonoverlapping, but additional studies will be needed to confirm the ability to safely use this combination in patients.

## Methods

Detailed methods can be found in Supplemental Methods.

### Sex as a biological variable.

Our study used both female and male immunodeficient NSG (NOD.Cg-Prkdcscid Il2rgtm1Wjl/SzJ) mice (IMSR catalog JAX:005557, RRID: IMSR_JAX:005557, The Jackson Laboratory), and similar findings are reported for both sexes.

### Animal studies.

NSG (NOD.Cg-Prkdcscid Il2rgtm1Wjl/SzJ) mice (IMSR catalog JAX:005557, RRID: IMSR_JAX:005557, The Jackson Laboratory) were used to assess GSC growth in vivo. In murine GBM experiments, C57BL/6 female mice (Jackson Laboratory) were used to assess the growth of CT2A mouse glioma cells in vivo. Briefly, both male and female mice aged from 4 to 6 weeks were randomly distributed to each group and maintained in a specific pathogen–free (SPF) animal facility on a 12-hour light/12-hour dark cycle at University of Pittsburgh. More details about drug treatment and intracranial tumor injection methods can be found in Supplemental Methods.

### GSC culture.

Patient-derived GSCs were derived from fresh GBM and cultured in Neurobasal media (Invitrogen, 12348017) supplemented with B27 (Invitrogen, 12587010), Glutamax (Invitrogen, 35050079), sodium pyruvate (Invitrogen, 11360070), penicillin-streptomycin (Invitrogen, 5140122), epidermal growth factor (rhEGF; 236-EG, R&D Systems), and basic fibroblast growth factor (bFGF; PHG0021, Thermo Fisher Scientific). Molecular profiling of GSCs is listed in Supplemental Table 1.

### Lentivirus production.

For lentivirus production, shRNA plasmids used in this paper were bought from Sigma. shRNA information is listed in Supplemental Table 2. Transfer plasmids were cotransfected with psPax2 (Addgene, 12260) and pMD2G (Addgene, 12259) using PEI (Polysciences, 24765-100) in HEK293T cells. Lentiviruses were collected on days 1, 2, and 3 after transfection.

### In vitro flow cytometry.

For in vitro flow cytometry analysis, GSCs were suspended in FACS buffer and stained with flow cytometry antibody for 30 minutes at room temperature. All the antibody information is listed in Supplemental Table 3. Doublets were excluded on the basis of side scatter area (SSC-A) and side scatter height (SSC-H) for flow cytometry analysis (Supplemental Figure 9).

### qRT-PCR.

RNA was extracted using the Direct-zol RNA Microprep Kit (Zymo Research, R2062), according to the manufacturer’s protocol. cDNA was synthesized with 1 μg RNA by reverse transcription using the High-Capacity cDNA Reverse Transcription Kit (Life Technologies, 4374966). Relative cDNA was quantified by performing qRT-PCR using Bio-Rad CFX 9600 with SYBR Green PCR Master Mix (Life Technologies, A25778). qRT-PCR primers for target genes and internal controls are listed in Supplemental Table 4.

### Statistics.

Detailed statistical methodology can be found in Supplemental Methods. All data are presented as mean ± SEM. The following statistical tests were used and are described in the figure legends: 2-tailed *t* test; 1-way ANOVA test with Dunnett’s multiple-comparisons test; 2-way ANOVA with correction by Dunnett’s multiple-comparisons test; and log-rank test. Statistical significance was set at *P* less than 0.05.

### Study approval.

All animal experiments were conducted under a protocol approved by Institutional Animal Care and Use Committee at the University of Pittsburgh.

### Data availability.

The data that support the findings of this study are available within the article and its supplemental tables and figures. Values for all data points in graphs are reported in the Supporting Data Values file. The RNA-Seq and ChIP-Seq data are deposited in the NCBI’s Gene Expression Omnibus database (GEO GSE245855).

## Author contributions

SW and JNR designed the overall experiments and analyzed data. Animal work was done by SW and QW. SW and TH performed flow cytometry experiments and data analysis. In vitro enzyme assays were performed by SW with the help of HY. SW performed bioinformatic analysis with the help of XW. SW and FY performed Western blot analysis. Immunofluorescence staining was done by SW with the help of TD in image analysis. YZ and NWS provided lactyl-CoA. SW, JNR, DGP, and ST wrote the manuscript.

## Supplementary Material

Supplemental data

Supplemental data set 1

Unedited blot and gel images

Supporting data values

## Version 1 11/15/2024

Electronic publication
