# Supplementary material for: Lactate reprograms glioblastoma immunity through CBX3-regulated histone lactylation
Source: J Clin Invest. 2024 Nov 15;134(22):e176851. doi: 10.1172/JCI176851 (PMC11563687; doi:10.1172/JCI176851)
Supplement: Supplemental data [file jci-134-176851-s113.pdf]

Supplemental Material

Lactate Reprograms Glioblastoma Immunity through CBX3-regulated Histone Lactylation

Shuai Wang[1,2], Tengfei Huang[1], Qiulian Wu[1], Huairui Yuan[1], Xujia Wu[1], Fanen Yuan[1], Tingting Duan[1], Suchet Taori[3], Yingming Zhao[4], Nathaniel W. Snyder[5], Dimitris G. Placantonakis[2], Jeremy N. Rich[1]

1. Hillman Cancer Center and Department of Neurology, University of Pittsburgh Medical Center, Pittsburgh, PA, USA
2. Department of Neurosurgery and Perlmutter Cancer Center, NYU Grossman School of Medicine, New York, NY, USA.
3. School of Medicine, University of Pittsburgh Medical Center, Pennsylvania, PA, USA.
4. Ben May Department for Cancer Research, The University of Chicago, Chicago, IL, USA.
5. Department of Cardiovascular Sciences, Lewis Katz School of Medicine at Temple University, Philadelphia, PA, USA.

Correspondence to: drjeremyrich@gmail.com or Dimitris.Placantonakis@nyulangone.org

The authors have declared that no conflict of interest exists.

Supplemental Material Content

Supplemental Figures S1-S7

Supplemental Methods

Supplemental Tables

Supplemental References

Supplemental Figures S1-S7

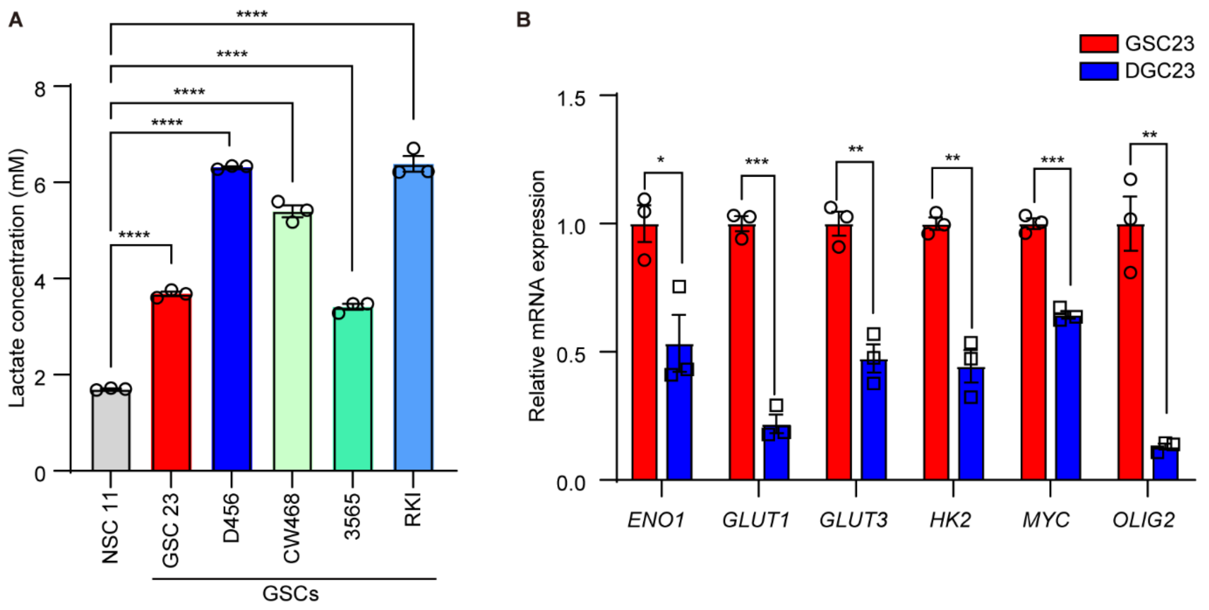

Supplemental Figure 1: Lactate levels are higher in GSCs compared to NSCs.

(**A**) Concentrations of lactate in the culture medium of NSCs (NSC11) and GSCs (GSC23, D456, CW468, 3565, and RKI) (n=3/group; one-way ANOVA, $F (5, 12) = 424.8$).

(**B**) qRT-PCR analysis of glycolysis-related genes in GSC23 and DGC23 cells. *TUBA1A* was used as the housekeeping gene control (n=3/group; t-tests).

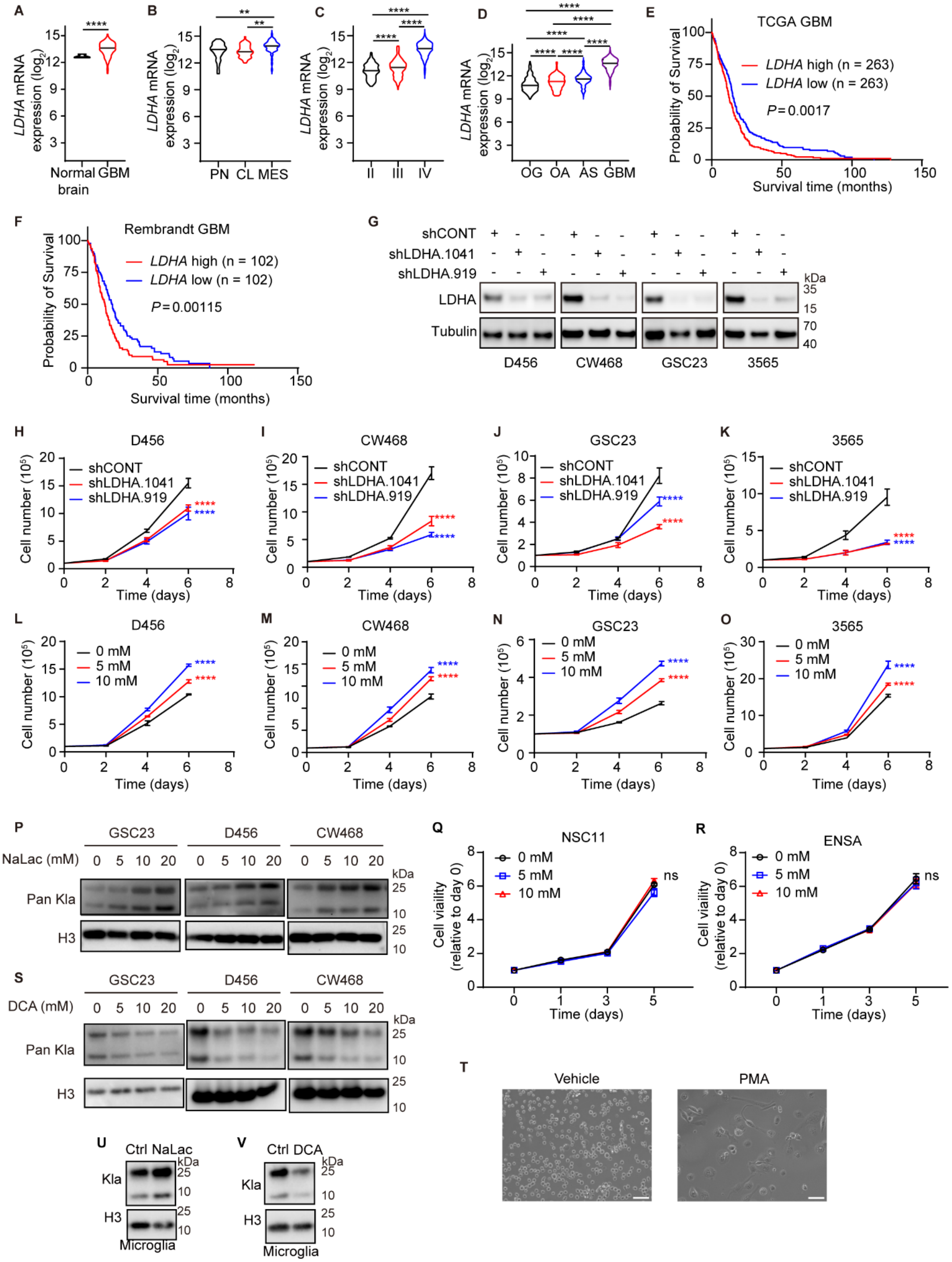

A
LDHA mRNA expression (log2)
Normal brain
GBM
B
PN CL MES
C
II III IV
D
OG OA AS GBM
E
TCGA GBM
LDHA high (n = 263)
LDHA low (n = 263)
P = 0.0017
Probability of Survival
Survival time (months)
F
Rembrandt GBM
LDHA high (n = 102)
LDHA low (n = 102)
P = 0.00115
G
shCONT
shLDHA.1041
shLDHA.919
LDHA
Tubulin
kDa
D456
CW468
GSC23
3565
H
I
J
K
Cell number (10^5)
Time (days)
L
M
N
O
0 mM
5 mM
10 mM
P
NaLac (mM)
Pan Kla
H3
Q
NSC11
R
ENSA
Cell viability (relative to day 0)
ns
S
DCA (mM)
T
Vehicle
PMA
U
Ctrl NaLac
Kla
Microglia
V
Ctrl DCA

Supplemental Figure 2: Lactate promotes GBM cell growth and immune evasion.

(**A**) mRNA expression (normalized count values) of *LDHA* in normal brain and GBM. TCGA data were downloaded from Gliovis.

(**B**) mRNA expression of *LDHA* in PN (proneural), CL (classical), and MES (mesenchymal) GBM. TCGA data were downloaded from Gliovis (one-way ANOVA; F (2, 153) = 7.127).

(**C**) mRNA expression of *LDHA* in different WHO grades of gliomas. TCGA data were downloaded from Gliovis (one-way ANOVA; F (2, 617) = 378.9).

(**D**) mRNA expression of *LDHA* in OG (oligodendroglioma), OA (oligoastrocytoma), AS (astrocytoma), and GBM (one-way ANOVA; F (3, 663) = 285.0).

(**E**, **F**) Kaplan-Meier survival curves of *LDHA*-low and -high expression patients in TCGA (**E**) and Rembrandt (**F**) GBM databases (log-rank tests).

(**G**) Western blot shows the protein levels of LDHA in D456, CW468, GSC23, and 3565 cells transduced with shCONT or shLDHA. TUBULIN was used as loading control.

(**H** - **K**) LDHA knockdown inhibits proliferation of D456 (**H**), CW468 (**I**), GSC23 (**J**) and 3565 (**K**) (two-way ANOVAs; F (6, 18) = 9.019 for D456, F (6, 18) = 33.86 for CW468, F (6, 18) = 19.33 in GSC23, F (6, 18) = 21.99 for 3565).

(**L** - **O**) Lactate promotes the proliferation of D456 (**L**), CW468 (**M**), GSC23 (**N**) and 3565 (**O**) (two-way ANOVAs; F (6, 18) = 45.09 for D456, F (6, 18) = 17.28 for CW468, F (6, 18) = 45.77 for GSC23, F (6, 18) = 30.54 for 3565).

(**P**) Western blot shows the level of histone lactylation in GSC23, D456, and CW468 treated with different concentrations of NaLac. H3 was used as loading control.

(**Q**, **R**) Human neural stem cell lines NSC11 (**Q**) and ENSA (**R**) were treated with either vehicle control (PBS) or 5 or 10 mM NaLac, then proliferation was measured over a time course by CellTiter-Glo Assays (two-way ANOVAs; $F(6, 27) = 0.3376$ for NSC11, $F(6, 27) = 3.963$ for ENSA).

(**S**) Western blot shows the level of histone lactylation in GSC23, D456, and CW468 treated with DCA. H3 was used as loading control.

(**T**) THP-1 monocytes were differentiated into macrophages by 48-hour incubation with 200 nM phorbol 12-myristate 13-acetate (PMA), followed by 72-hour incubation in RPMI medium. Scale bar = 20 µm.

(**U**) Human microglia were cultured with either vehicle control (PBS) or NaLac (10 mM) for 24 hours. Whole cell lysates were collected and resolved by SDS-PAGE. Lactylated histones were measured by western blot. H3 was used as loading control.

(**V**) Human microglia were cultured with either vehicle control (PBS) or DCA (10 mM) for 24 hours. Whole cell lysates were collected and resolved by SDS-PAGE. Lactylated histones were measured by western blot. H3 was used as loading control.

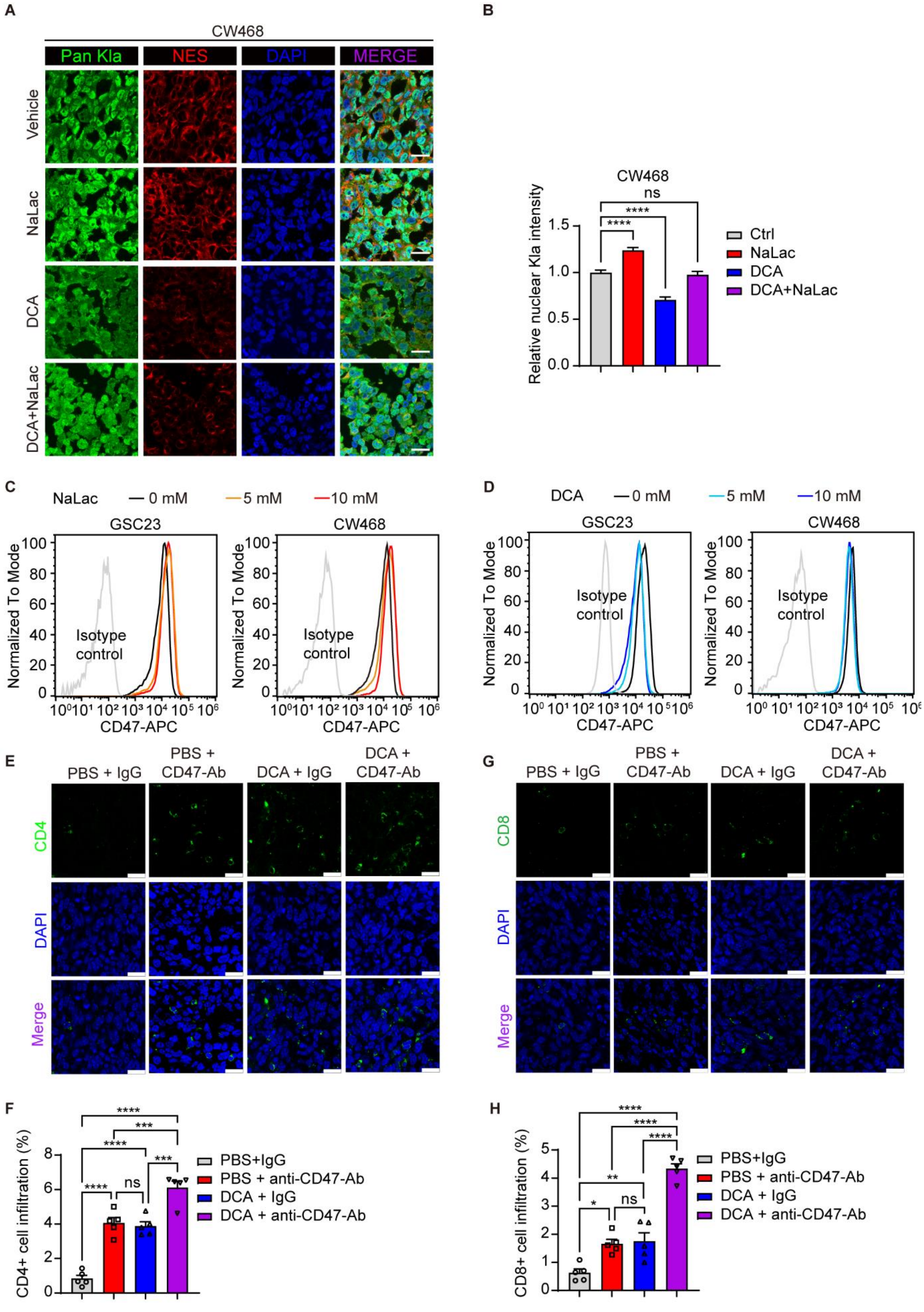
A
CW468
Pan Kla
NES
DAPI
MERGE
Vehicle
NaLac
DCA
DCA+NaLac
B
CW468
ns
****
****
Relative nuclear Kla intensity
Ctrl
NaLac
DCA
DCA+NaLac
C
NaLac
0 mM
5 mM
10 mM
GSC23
CW468
Normalized To Mode
Isotype control
CD47-APC
D
DCA
0 mM
5 mM
10 mM
GSC23
CW468
Normalized To Mode
Isotype control
CD47-APC
E
PBS + IgG
PBS + CD47-Ab
DCA + IgG
DCA + CD47-Ab
CD4
DAPI
Merge
G
PBS + IgG
PBS + CD47-Ab
DCA + IgG
DCA + CD47-Ab
CD8
DAPI
Merge
F
CD4+ cell infiltration (%)
PBS+IgG
PBS + anti-CD47-Ab
DCA + IgG
DCA + anti-CD47-Ab
H
CD8+ cell infiltration (%)
PBS+IgG
PBS + anti-CD47-Ab
DCA + IgG
DCA + anti-CD47-Ab

Supplemental Figure 3: Histone lactylation promotes immunosuppression in the tumor microenvironment.

(**A**) Representative immunofluorescence images of histone lactylation in CW468 intracranial tumor xenografts treated with PBS vehicle, NaLac (1 g/kg/day), DCA (150 mg/kg/day) or DCA (150 mg/kg/day) + NaLac (1 g/kg/day). Human NESTIN marks tumor cells. DAPI marks nuclei. Scale bar = 20 μm.

(**B**) Statistical analysis of nuclear histone lactylation levels in CW468 intracranial tumor xenograft in (**A**) (n=37-58/group; one-way ANOVA; F (3, 199) = 50.95).

(**C, D**) Representative flow cytometry plots for CD47 expression in GSC23 and CW468 treated with different concentrations of NaLac (**C**) and DCA (**D**).

(**E**) Immunofluorescence staining of CD4 in brain slices of tumor-bearing mice implanted with CT2A cells and treated with PBS + control IgG (100 µg/mouse), PBS + anti-CD47-Ab (100 µg/mouse), DCA (150 mg/kg/day) + IgG (100 µg/mouse), or DCA (150 mg/kg/day) + CD47-Ab (100 µg/mouse). DAPI marks nuclei. Scale bar = 20 μm.

(**F**) Quantification of CD4 cell infiltration in (**E**) (n=5 mice/group; one-way ANOVA; F (3, 16) = 56.09).

(**G**) Immunofluorescence staining of CD8 in brain slices of tumor-bearing mice implanted with CT2A cells and treated with PBS + IgG (100 µg/mouse), PBS + anti-CD47-Ab (100 µg/mouse), DCA (150 mg/kg/day) + IgG (100 µg/mouse), or DCA (150 mg/kg/day) + anti-CD47-Ab (100 µg/mouse). DAPI was used to label nuclei. Scale bar = 20 µm.

(**H**) Quantification analysis of CD8 cell infiltration in (**G**) (n=5 mice/group; one-way ANOVA; $F(3, 16) = 61.81$).

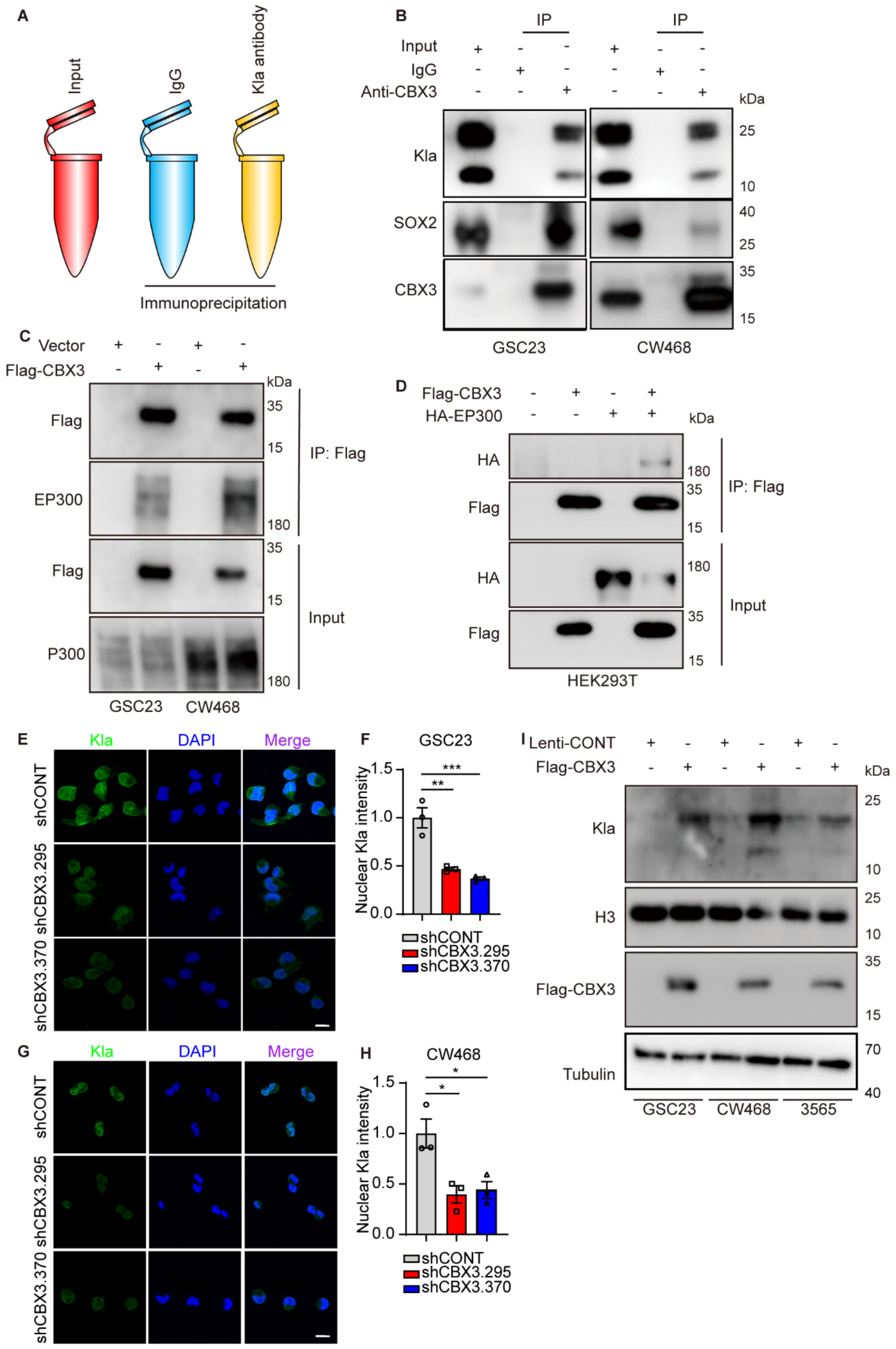
A
Input
IgG
Kla antibody
Immunoprecipitation
B
IP
Input
IgG
Anti-CBX3
kDa
Kla
SOX2
CBX3
GSC23
CW468
C
Vector
Flag-CBX3
Flag
EP300
P300
IP: Flag
Input
D
HA-EP300
HA
HEK293T
E
Kla
DAPI
Merge
shCONT
shCBX3.295
shCBX3.370
F
Nuclear Kla intensity
G
H
I
Lenti-CONT
H3
Tubulin
3565

Supplemental figure 4: Knockdown of CBX3 decreases histone lactylation in GBM cells.

(**A**) Schematic shows the design of immunoprecipitation analysis.

(**B**) Co-IP showing the interaction between Kla, SOX2, and CBX3 in GSC23 and CW468 cells.

(**C**) Co-IP analysis of the interaction between FLAG-tagged CBX3 and EP300 in GSC23 and CW468 cells transduced with either a control vector or flag-tagged CBX3. Immunoprecipitation was performed with M2 beads.

(**D**) Co-IP of the interaction between FLAG-CBX3 and HA-EP300 in HEK293T cells. Immunoprecipitation was performed with M2 beads.

(**E**) Immunofluorescence staining of histone lactylation in GSC23 transduced with shCONT or shCBX3. Scale bar = 20 μm.

(**F**) Quantification of nuclear histone lactylation staining in GSC23 transduced with shCONT or shCBX3 (n = 3/group; one-way ANOVA; F (2, 6) = 29.95).

(**G**) Immunofluorescence staining of histone lactylation in CW468 transduced with shCONT or shCBX3. Scale bar = 20 µm.

(**H**) Quantification of nuclear histone lactylation staining in GSC23 transduced with shCONT or shCBX3 (n = 3/group; one-way ANOVA; F (2, 6) = 9.847).

(**I**) Western blot shows the levels of histone lactylation in GSC23, CW468, and 3565 cells transduced with lentiviral-CONT (empty vector) or FLAG-CBX3. H3 and TUBULIN were used as loading controls.

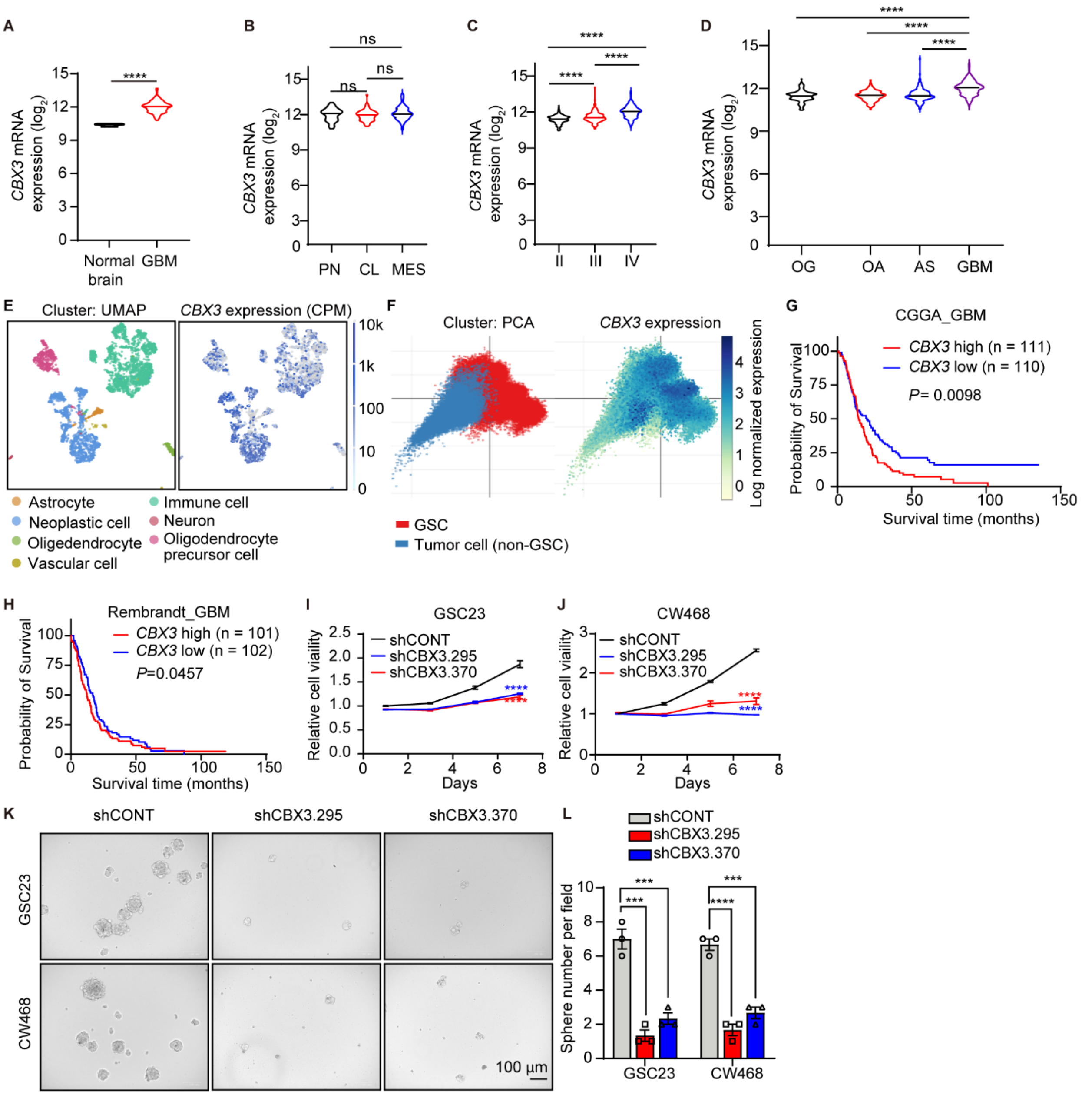

Supplemental figure 5: CBX3 expression and tumorigenic effects in GBM.

(**A**) mRNA expression (normalized count values) of *CBX3* in normal brain and GBM. TCGA data were downloaded from Gliovis.

(**B**) mRNA (normalized count values) expression of *CBX3* in PN (proneural), CL (classical), and MES (mesenchymal) GBM. TCGA data were downloaded from Gliovis (one-way ANOVA; F (2, 153) = 1.617).

(**C**) mRNA expression (normalized count values) of *CBX3* in different WHO grades of gliomas. TCGA data were downloaded from Gliovis (one-way ANOVA; F (2, 617) = 98.05).

(**D**) mRNA expression (normalized count values) of *CBX3* in OG, OA, AS, and GBM. (one-way ANOVA; F (3, 663) = 63.20).

(**E**) UMAP visualization of *CBX3* expression in 3588 cells (GSE84465) using Single Cell Expression Atlas (https://www.ebi.ac.uk/gxa/sc/home). Different cell types are annotated.

(**F**) PCA visualization of *CBX3* expression in GSCs (glioma stem cells) and non-GSC tumor cells using Broad Institute Single-Cell Portal (https://singlecell.broadinstitute.org/single_cell, SCP503). GSCs were characterized by high expression of stem-like markers, while differentiated tumor cells (non-GSCs) were characterized by high expression of mature astrocyte markers (1).

(**G,H**) Kaplan-Meier survival curves of *CBX3*-low and *CBX3*-high patients in the CGGA (**G**) and Rembrandt (**H**) databases (log-rank tests).

(**I**, **J**) CBX3 knockdown inhibits proliferation of GSC23 (**I**) and CW468 (**J**) (n=4/group; two-way ANOVAs; F (6, 18) = 32.18 for GSC23, F (6, 18) = 140.9 for CW468).

(**K**) GSC23 and CW468 tumorsphere formation assay after transduction with either shCONT or shCBX3. Images were captured with bright field microscopy. Scale bar = 100 μm.

(**L**) Quantification of the sphere formation ability of GSC23 and CW468 cells transduced with either shCONT or shCBX3 (n=3/group; one-way ANOVAs; $F(2, 6) = 49.40$ for GSC23, $F(2, 6) = 63.00$ for CW468).

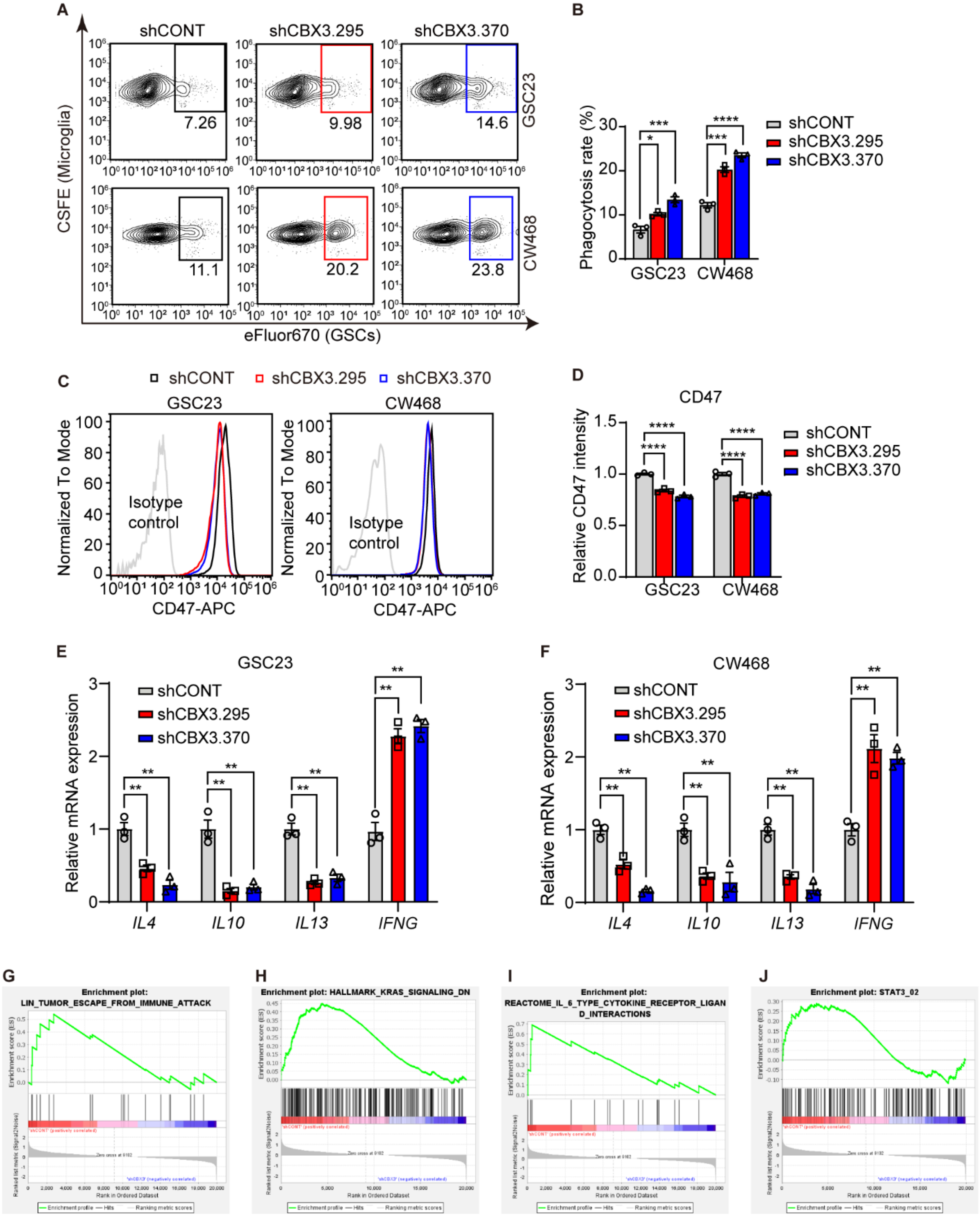
A
shCONT
shCBX3.295
shCBX3.370
7.26
9.98
14.6
GSC23
11.1
20.2
23.8
CW468
CSFE (Microglia)
eFluor670 (GSCs)
B
Phagocytosis rate (%)
GSC23
CW468
shCONT
shCBX3.295
shCBX3.370
C
GSC23
CW468
Normalized To Mode
Isotype control
CD47-APC
D
CD47
Relative CD47 intensity
E
GSC23
Relative mRNA expression
IL4
IL10
IL13
IFNG
F
CW468
G
Enrichment plot: LIN_TUMOR_ESCAPE_FROM_IMMUNE_ATTACK
H
Enrichment plot: HALLMARK_KRAS_SIGNALING_DN
I
Enrichment plot: REACTOME_IL_6_TYPE_CYTOKINE_RECEPTOR_LIGAND_INTERACTIONS
J
Enrichment plot: STAT3_02
Enrichment score (ES)
Rank in Ordered Dataset

Supplemental figure 6: CBX3 regulates the immune response in GBM.

(**A**) Representative flow cytometric assessment of phagocytosis of GBM cells (stained witheFluor670) by microglia (stained with CSFE). GBM cells were transduced with either shCONT or shCBX3.

(**B**) Quantification of phagocytosis rates in (**A**) (n = 3/group; one-way ANOVA; F (2, 6) = 29.61 for GSC23), F (2, 6) = 100.5 for CW468).

(**C**) Representative flow cytometry plots for CD47 expression in GSC23 and CW468 transduced with either shCONT or shCBX3.

(**D**) Quantification of CD47 expression (median fluorescence intensity) recorded with flow cytometry in (**C**) (n = 3/group; one-way ANOVA; F (2, 6) = 104.4 for GSC23, F (2, 6) = 91.13 for CW468).

(**E**, **F**) qRT-PCR analysis of *IL4*, *IL10*, *IL13*, and *IFNG* in GSC23 and CW468 transduced with either shCONT or shCBX3. *ACTB* was used as housekeeping gene control. (n=3/group; one-way ANOVAs; F (2, 6) = 34.19 for *IL4*, F (2, 6) = 37.20 for *IL10*, F (2, 6) = 51.54 for *IL13*, F (2, 6) = 57.46 for *IFNG*, in GSC23 cells; F (2, 6) = 63.52 for *IL4*, F (2, 6) = 16.91 for *IL10*, F (2, 6) = 53.62 for *IL13*, F (2, 6) = 22.37 for *IFNG*, in CW468 cells).

(**G**-**J**) GSEA analysis of differentially expressed genes following CBX3 knockdown shows the top gene sets.

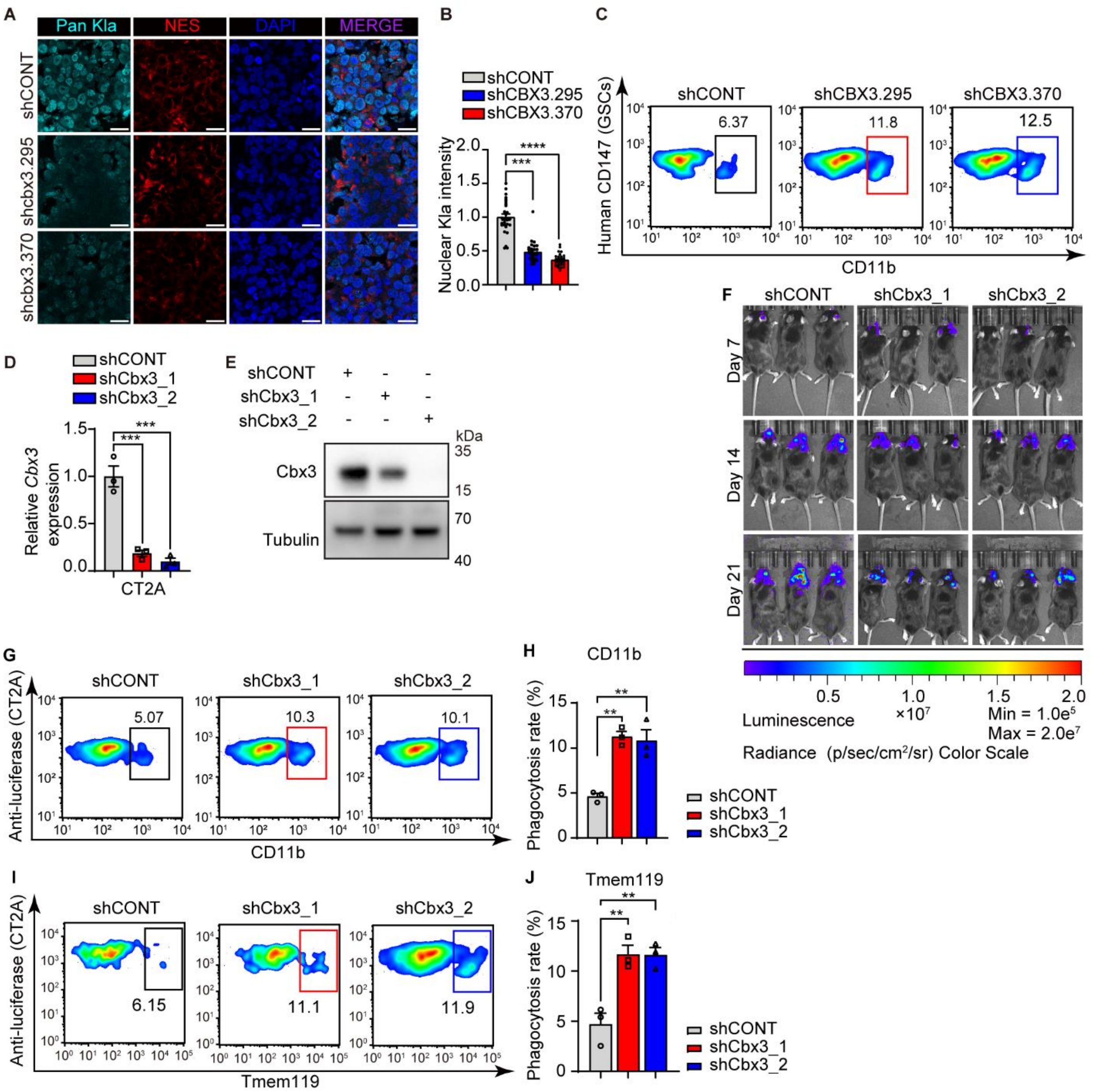

Supplemental figure 7: CBX3 regulates phagocytosis in vivo through histone lactylation.

(**A**) Immunofluorescence staining of histone lactylation in CW468 intracranial tumor xenografts transduced with either shCONT or shCBX3. Human NESTIN marks tumor cells. DAPI marks nuclei. Scale bar = 20 μm.

(**B**) Statistical analysis of nuclear histone lactylation levels in CW468 intracranial tumor xenograft transduced with either shCONT or shCBX3 (n=37-58/group; one-way ANOVA; F (2, 87) = 116.5).

(**C**) Representative flow cytometry plots of in vivo phagocytosis in CW468 intracranial tumor xenografts transduced with either shCONT, shCBX3.295, or shCBX3.370 viruses. CD11b was used to label microglia/macrophage. Human CD147 was used to label GBM cells.

(**D**) qRT-PCR analysis of murine *Cbx3* in CT2A cells transduced with either shCONT or shCbx3. *Actb* was used as internal control (n=3/group; one-way ANODA; F (2, 6) = 51.08).

(**E**) Western blot of Cbx3 in CT2A cells transduced with shCONT or shCbx3. Tubulin was used as loading control.

(**F**) Representative bioluminescent images on days 7, 14, and 21 of mice intracranially implanted with CT2A cells transduced with shCONT or shCbx3.

(**G**) Representative flow cytometry plots of in vivo phagocytosis in CT2A murine gliomas. CT2A cells were transduced with control shRNA (shCONT) or mouse Cbx3 knockdown shRNA (shCbx3_1 or shCbx3_2). Microglia were labeled by CD11b. CT2A cells were labeled by luciferase.

(**H**) Quantification of in vivo phagocytosis in (**G**) (n = 3/group; one-way ANOVA; F (2, 6) = 21.53).

(**I**) Representative flow cytometry plots of in vivo phagocytosis in CT2A murine gliomas. CT2A cells were transduced with control shRNA (shCONT) or mouse Cbx3 knockdown shRNA (shCbx3_1 or shCbx3_2). Microglia were labeled by TMEM119. CT2A cells were labeled by luciferase.

(**J**) Graphic quantification of in vivo phagocytosis in (**I**) (n = 3/group; one-way ANOVA; F (2, 6) = 18.70).

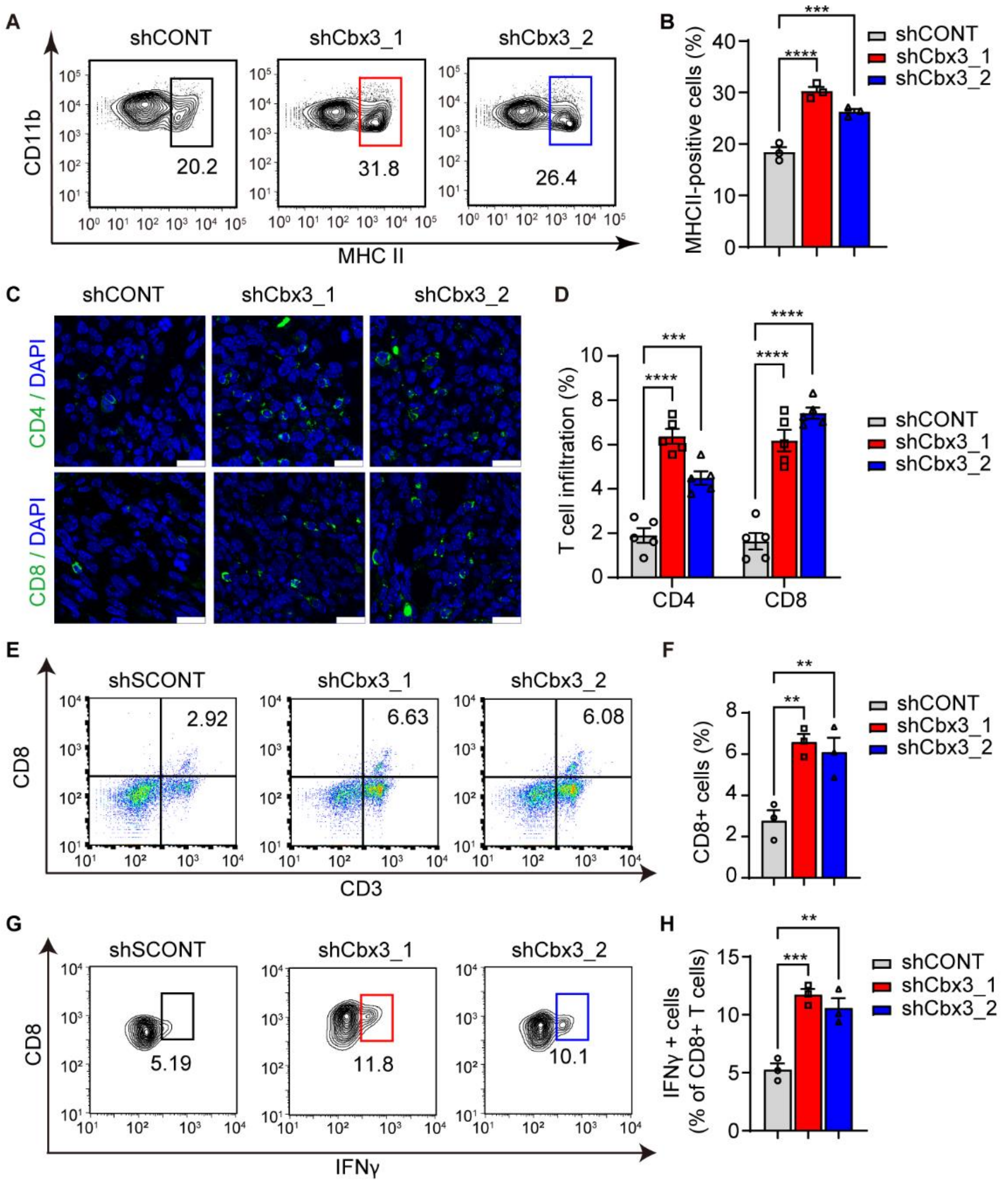

Supplemental figure 8: CBX3 regulates the immune response in CT2A gliomas.

(**A**) Representative flow cytometry plots of MHCII-positive microglia in tumor-bearing murine brains implanted with CT2A cells. CT2A cells were transduced with either a non-targeting control shRNA sequence (shCONT) or one of two, non-overlapping shRNAs targeting mouse Cbx3 (shCbx3_1 or shCbx3_2).

(**B**) Quantification of MHCII-positive microglia in tumor-bearing mouse brains implanted with CT2A cells transduced with control shRNA (shCONT) or shRNA targeting murine Cbx3 (n=3/group; one-way ANOVA; $F(2, 6) = 55.46$).

(**C**) Immunofluorescence staining of CD4+ and CD8+ cells using brain slices of tumor-bearing mice implanted with CT2A cells transduced with shCONT or shCbx3. DAPI marks nuclei. Scale bar = 20 μm.

(**D**) Quantification of CD4+ and CD8+ cell tumor infiltration in mice implanted with CT2A cells transduced with shCONT or shCbx3 (n = 5/group; one-way ANOVAs; $F(2, 12) = 48.08$ for CD4, $F(2, 12) = 62.43$ for CD8).

(**E**) Representative flow cytometry plots of CD8+ T lymphocyte infiltration in tumor-bearing mice intracranially implanted with CT2A cells transduced with either control shRNA (shCONT) or shRNA targeting murine Cbx3.

(**F**) Quantification of CD8+ T lymphocyte infiltration in tumor-bearing brain implanted with CT2A cells transduced with non-targeting control shRNA (shCONT) or shRNA targeting murine Cbx3 (n = 3/group; one-way ANOVA; $F(2, 6) = 14.13$).

(**G**) Representative flow cytometry plots of IFNγ+ / CD8+ T lymphocyte infiltration in CT2A allografts transduced with either control shRNA (shCONT) or shRNA targeting mouse Cbx3.

(**H**) Quantification of IFNγ+ / CD8+ T lymphocyte infiltration in tumor-bearing brain implanted with CT2A cells transduced with control shRNA (shCONT) or shRNA targeting murine Cbx3 (n = 3/group; one-way ANOVA; $F(2, 6) = 27.68$).

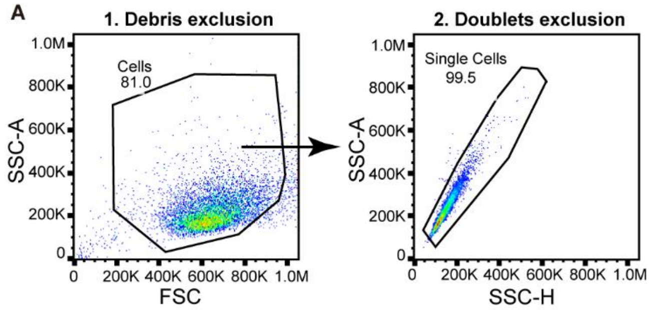

Supplemental figure 9: Representative example of the method used to exclude debris and doublets in flow cytometry. The first gate excluded debris based on forward and side scatter (FSC and SSC). Following this, doublets were excluded on the basis of side scatter area (SSC-A) and side scatter height (SSC-H).

Supplemental Methods

Cell lines and cell culture

Patient-derived GBM stem cells (GSCs) were derived from fresh GBM specimens as described (2), then functionally confirmed through a series of assays, including orthotopic implantation in immunodeficient NSG (NOD.Cg-*Prkdc*^{scid} *Il2rg*^{tm1Wjl}/SzJ) mice (IMSR Cat# JAX:005557, RRID: IMSR_JAX:005557, The Jackson Laboratory, Bar Harbor, ME) to verify their *in vivo* tumorgenicity (for molecular profiling, see Supplemental Table 1). Three preparations of human neural precursor cells (NPCs), including ENSA (ENStem-A, Millipore Sigma, catalog no. SCC003), hNP1 (Neuromics, catalog no. HN60001), and NSC11 (Alstem, catalog no. hNSC11), were used in our study. ENSA cells were induced towards a neural lineage from human embryonic stem cells. hNP1 human progenitors were fully differentiated and derived as adherent cells from hESC WA09 line. NSC11 cells were derived from human induced pluripotent stem cells, which were generated from adult skin fibroblasts. All GSCs and NPCs were cultured in Neurobasal media (Invitrogen, 12348017) supplemented with 20 µl/ml B27 (Invitrogen, 12587010), 1% Glutamax (Invitrogen, 35050079), 1 mM sodium pyruvate (Invitrogen, 11360070), 1% penicillin-streptomycin (Invitrogen, 5140122), 20 ng/ml epidermal growth factor (rhEGF; 236-EG, R&D Systems), and 20 ng/ml basic fibroblast growth factor (bFGF; PHG0021, Thermo Fisher Scientific) at 37°C with 20% oxygen and 5% carbon dioxide.

Differentiated GBM cells (DGCs) were derived from GSCs by adding 10% fetal bovine serum (FBS; Invitrogen, A4766801) to DMEM medium (Invitrogen, 11965092) for at least 1 week. Microglia,

CT2A, HEK293T, and DGCs were cultured in DMEM medium supplemented with 10% FBS, 1 mM sodium pyruvate (Invitrogen, 11360070), and 1% penicillin-streptomycin (Invitrogen, 5140122). THP1 cells were cultured in RPMI 1640 (Corning, 10-040-CV) medium supplemented with 10% FBS and penicillin-streptomycin (Invitrogen, 5140122). THP-1 monocytes were differentiated into macrophages by 48 h incubation with 200 nM phorbol 12-myristate 13-acetate (PMA, MCE, HY-18739) followed by 72 h incubation in RPMI medium, as previously described (3).

Lentivirus production and transduction

For lentivirus production, shRNA plasmids used in this paper were bought from Sigma. shRNA information is listed in Supplemental Table 2. Flag-tagged CBX3 plasmid for lentivirus production was bought from Vector Builder. Transfer shRNA plasmids or Flag-tagged CBX3 plasmids were co-transfected with psPax2 (Addgene, 12260) and pMD2G (Addgene, 12259) using PEI (Polysciences, 24765-100) in HEK293T cells. Lentiviruses were collected on days 1, 2, and 3 after transfection, followed by twenty-fold concentration with Lenti-X Concentrator (Takara, 631232). For lentiviral transduction of GSCs, GBM cells were dissociated into single cells using Accutase (Thermo Fisher, 00-4555-56) before transduction. In brief, GSCs were plated into 6-well plates with 3-5 μL concentrated lentivirus at the density of 300,000 cells per well. The culture medium was changed 24 hours after transduction, and 2 ng/μL puromycin was added for selection of lentivirus-transduced cells 72 hours after transduction. GSCs were cultured for at least one week in puromycin-containing culture medium to eliminate non-transduced cells. All the viruses used in the study were lentiviruses.

Co-culture of microglia and GSCs

Before co-culture, microglia were treated with 100 ng/mL LPS (MCE, HY-D1056) for 24 hours to induce an M1-like phenotype (4). We induced M2-like cell states in microglia using either interleukin-4 (10 ng/ml IL4, MCE, HY-P70445; designated as M2-IL4) or interleukin-13 (10 ng/ml IL13, MCE, HY-P72795; designated as M2-IL13) for 24 hours (4). For co-culture experiments intended to assess lactate production by microglia, non-polarized or polarized microglia were plated on culture inserts (Millipore, TMO140640) in 6-well plates at a density of 3 x 10^5 cells per insert, and 3 x 10^5 GBM cells/well were plated in 6-well plates. After 48 hours, GBM cells were harvested for western blot analysis and lactate measurement assays.

Lactate measurement assay

Lactate concentrations in culture media or cytoplasm were measured using Lactate-Glo™ Assay kit (Promega, J5021) according to the technical manual. In brief, culture medium was collected followed by fifty-fold dilution in PBS. Fifty µl were transferred to a 96-well plate (Corning, 3917), followed by 50 µl of Lactate Detection Reagent, prepared as recommended by the manufacturer. Plates were shaken for 30 seconds, then incubated for 60 minutes at room temperature before recording luminance. To measure cytosolic lactate contents, 50,000 cells were collected followed by washing twice with PBS (phosphate-buffered saline). Washed cells were resuspended in 25 µl PBS followed by 12.5 µl of Inactivation Solution, 12.5 µl of Neutralization Solution, and 50 µl of Lactate Detection Reagent prepared as recommended. Plates were shaken for 30 seconds, then

incubated for 60 minutes at room temperature before recording luminance. All luminance values were normalized to control groups.

Glycolysis stress test

The Glycolysis stress test was measured using Agilent Seahorse XF and Glycolysis Stress Test Kit (Agilent, 103017-100). In brief, 10,000 GSCs or DGCs were plated in Seahorse XF Microplates and a sensor cartridge was hydrated in Seahorse XF Calibrant at 37°C in a non-CO_2 incubator overnight. Glucose, oligomycin, and 2-DG (2-Deoxy-D-Glucose) were loaded into the appropriate ports of a hydrated sensor cartridge. Culture media were changed into pre-warmed assay media, and the cell culture microplate was placed into a 37 °C non-CO_2 incubator for 45 minutes to 1 hour prior to the assay. The machine injected the drugs into the assay media sequentially. After data collection, raw data were exported via Excel and plotted using GraphPad Prism.

Cell growth and sphere formation assays

Cell growth was measured in 96-well plates by using CellTiter-Glo 2.0 (Promega, G9242) according to the manufacturer's protocol. Briefly, 2×10^3 cells were seeded per well in 96-well plates with at least 3 technical replicates. Then CellTiter-Glo 2.0 was added to the wells at desired time points and transferred to black plates to measure the luminescence. All the measurements were normalized to luminescence values at day 0 and presented as mean ± standard error of the mean (SEM). For sphere formation assays, 500 cells were seeded into 96-well plates. Each well

was cultured for 2 weeks and then images were captured under the bright field of an optical microscope.

Limiting dilution assay

GSCs were seeded into 96-well plates with 10 replicates at multiple numbers of cells per well (200, 100, 50, 20, 10, or 1 cell/well). The number of wells with spheres was recorded on day 14. Data were visualized by using online software: http://bioinf.wehi.edu.au/software/elda/.

Immunofluorescent staining

GSCs were dissociated into single cells and plated on Matrigel (Corning, 354227)-treated coverslips for 2 days. For murine intracranial xenografts, brains were fixed with 4% PFA for 3 days and dehydrated in 30% sucrose for 2 days followed by embedding in OCT (Scigen, 4586). Fifteen µm brain slices were sectioned using a rotary microtome (Leica, CM1850-3-1). Cells or tissues sections were washed with PBS (phosphate-buffered saline) followed by fixation with 4% paraformaldehyde and permeabilization with 0.1% PBST (PBS with Triton-100) for 10 minutes. After blocking with 5% normal goat serum in 0.05% PBST (PBS with Tween 20) for 30 minutes at room temperature, antibodies were diluted according to manufacturer's instructions and incubated at 4°C overnight. Coverslips with cells or tissue sections were washed with 0.05% PBST 3 times for 5 minutes each, followed by incubation with secondary antibodies conjugated with desired fluorescent dyes for 2 hours at room temperature. Finally, nuclei were stained with DAPI

for 15 minutes followed by rinsing with 0.05% PBST 3 times for 5 minutes each. Antifade Mountant (Invitrogen, S36917) was applied to prevent fluorescence photobleaching before capturing images with confocal microscopy (Leica, SP8). All the pictures captured were processed with FIJI (https://imagej.net/software/fiji/). All antibodies used in this study are listed in Supplemental Table 3.

Co-immunoprecipitation and western blotting

Cells were lysed by RIPA lysis buffer (Thermo Fisher, 89901) supplemented with protease inhibitor (Thermo Fisher, A32963) and phosphatase inhibitor (Thermo Fisher, A32957). Cell lysates were mixed by rotation at 4°C for 15 minutes followed by centrifugation at 17,000 g for 10 minutes at 4°C. The supernatant was transferred to new tubes and processed for protein concentration quantification by Bradford assay (BioRad, 5000002). For co-immunoprecipitation experiments, 10 µg protein was used as input (the total protein used as positive control) and 1 mg protein diluted with IP buffer (Thermo Fisher, 87787) to 500 µL was used for immunoprecipitation. Diluted lysates were added to 20 µL protein A/G magnetic beads (ThermoFisher, 88802) pre-incubated with IgG or specific antibodies for 2 hours at 4°C, then incubated with rotation at 4°C overnight. After that, beads were rinsed with IP buffer 3 times, each for 5 minutes at 4°C, followed by elution with LDS buffer (Thermo Fisher, NP0007) at 90°C for 10 minutes. For western blotting, protein lysates were denatured with LDS buffer (Thermo Fisher, NP0007) at 90°C for 10 minutes, and 10 µg protein of each sample was resolved by SDS PAGE. Proteins were transferred from gels to PVDF membranes and blocked with defatted milk

for 1 hour at room temperature, followed by incubation with desired antibodies (See Supplemental Table 3) at 4°C overnight. After washing with TBST (Tris-buffered saline with 0.1% Tween 20) 3 times (10 minutes each at room temperature), the PVDF membrane was incubated with HRP-conjugated secondary antibodies for 1 hour. HRP signals were developed by using SuperSignal™ West Dura Extended Duration Substrate (Thermo Fisher, 34076) and captured by ChemiDoc Imaging System (BioRad). To derive optimal comparisons of protein levels by western blot, we captured multiple images with different exposure times to optimize detection of values in the linear dynamic range.

In vitro acetylation and lactylation assays

Lactyl-CoA was kindly provided by Dr. Yingming Zhao. Histone octamer (SRP0408), HAT Assay Buffer (Millipore, 20-148), CBX3 recombinant protein (Novus Biologicals, NBP1-99065), and P300 recombinant protein (BPS Bioscience, 50071) were mixed with 1 mM lactyl-CoA or acetyl-CoA (MCE, HY-113596) on ice followed by incubation at 37°C for 20 minutes. Reaction products were denatured with LDS buffer (Thermo Fisher, NP0007) at 90°C for 10 minutes for western blotting.

RNA-isolation and qRT-PCR

Cells were lysed with Trizol (Invitrogen, 15596026) and RNA was extracted with Direct-zol RNA Microprep kit (Zymo Research, R2062), according to the manufacturer's protocol. cDNA was synthesized with 1 μg RNA by reverse transcription using High-Capacity cDNA Reverse

Transcription kit (Life Technologies, 4374966). Relative cDNA was quantified by performing qRT-PCR using Bio-Rad CFX 9600 with SYBR Green PCR Master Mix (Life Technologies, A25778). qRT-PCR primers for target genes and internal controls are listed in Supplemental Table 4.

Flow cytometry

For in vitro flow cytometry analysis of CD47 expression, GSCs were washed with cold DPBS (Dulbecco's PBS; Thermo Fisher, 14190144) 3 times. 200,000 cells were suspended in FACS buffer containing 3% BSA and stained with APC-conjugated CD47 Antibody (Biolegend, 323123) for 30 minutes at room temperature. Cells were washed with FACS buffer for 3 times before flow cytometry analysis. Finally, cells were resuspended in 300 μL FACS buffer for flow cytometry analysis. Doublets were excluded on the basis of side scatter area (SSC-A) and side scatter height (SSC-H) (Supplemental Figure 9).

For in vivo flow cytometry analysis, mice were perfused with 0.9% saline to eliminate red blood cells. The whole brain with tumor xenografts was minced with blades and dissociated into single cells using collagenase I (Gibco, 17100017) at 37°C for 15 minutes. Myelin sheath was removed by percoll (Sigma, P1644-100ML) gradient centrifugation. Stock isotonic percoll (SIP) was prepared by mixing 9 parts of percoll with one part of 10 X HBSS without Ca^{2+} and Mg^{2+} (Sigma). SIP was added to cells suspended in PBS to make a final 30% SIP solution. The cell suspension in 30% SIP was then layered on top of a 70% SIP solution followed by centrifugation for 30 minutes at 500 g with minimal brake. Cells were fixed with 2% PFA for 15 minutes to preserve the

architecture after enzymatic dissociation. Further, cells were permeabilized with 0.1% Triton X-100 for 10 minutes before staining. Cells were suspended in FACS buffer containing 3% BSA and stained with FITC-conjugated anti-human CD147 Antibody (Biolegend, 306204) and PE anti-mouse/human CD11b Antibody (Biolegend, 101208) to analyze phagocytosis by flow cytometry in patient-derived GBM xenografts.

To analyze phagocytosis in the syngeneic mouse model, CT2A cells transduced with luciferase were identified with Recombinant Alexa Fluor® 647 Anti-Firefly Luciferase antibody (Abcam, ab237252), while microglia were identified with PerCP-eFluor™ 710 anti-TMEM119 (Thermo Fisher, 46-6119-82) or PE anti-mouse/human CD11b Antibody (Biolegend, 101208). To identify CD8 T cells, we used Alexa Fluor® 647 anti-mouse CD45 Antibody (Biolegend, 160303), FITC anti-mouse CD3 Antibody (Biolegend, 100203), and Spark UV™ 387 anti-mouse CD8a Antibody (Biolegend, 100797). In addition to antibodies used in CD8 T cell identification, we added PE anti-mouse IFN-γ Antibody (Biolegend, 505807) to quantify the expression of IFN-γ in T cells in the CT2A GBM mouse model. To quantify the expression of MHC II in microglia, we used PE anti-mouse/human CD11b Antibody (Biolegend, 101208) and APC anti-mouse MHC II Antibody (Biolegend, 107613).

In vitro phagocytosis assay

Microglia or macrophage were stained with carboxyfluorescein succinimidyl ester (CSFE; Thermo Fisher, C34570) in DMEM for 20 minutes at room temperature with gentle agitation. CSFE

staining was quenched by a brief wash with complete microglia culture medium containing 10% FBS. GSCs were stained with eBioscience™ Cell Proliferation Dye eFluor™ 670 (Thermo Fisher, 65-0840-85) in Neurobasal medium for 20 minutes at room temperature, followed by quenching with a brief wash using Neurobasal medium containing 3% BSA. CSFE-labeled microglia or macrophage were plated at the density of 5×10^4 cells per well and allowed to adhere for 2 hours in 24-well plates or coverslips treated with Matrigel (Corning, 354227) for 1 hour. Adherent microglia/macrophage were co-cultured with 5×10^4 GSCs labeled with eBioscience™ Cell Proliferation Dye eFluor™ 670 (Thermo Fisher, 65-0840-85) in complete GBM culture medium for another 4 hours. Finally, for flow cytometry analysis of phagocytosis, cells were detached by Accutase (Thermo Fisher, 00-4555-56) after washing out floating cells with DPBS (Dulbecco's PBS; Thermo Fisher, 14190144). Phagocytosis was determined using Attune NxT Flow Cytometer (Thermo Fisher) and analyzed by FlowJo™ Software (BD Bioscience). For analysis of phagocytosis with imaging, cells on the coverslips were fixed with 4% PFA and mounted using Antifade Mountant (Invitrogen, S36917) followed by imaging with a confocal microscope (Leica SP8). Three-dimensional (3D) reconstruction of phagocytosis was performed and analyzed using ImageJ.

Mass spectrometry

For protein mass spectrometry (MS) analysis, total proteins from GSC23 were harvested in RIPA buffer. Pierce™ BCA Protein Assay Kit (Thermo Scientific, 23225) was used to quantify protein concentration. One mg protein was diluted with IP buffer and used for the following

immunoprecipitation. Diluted lysate was added to 20 µL protein A/G magnetic beads (ThermoFisher, 88802) pre-incubated with Normal Rabbit IgG (CST, 2729), or Anti-L-lactyl-lysine rabbit mAb (PTM Biolab, PTM-1401 RM) for 2 hours at 4°C, then incubated with rotation at 4°C overnight. After that, beads were rinsed with IP buffer 3 times, each for 5 minutes at 4°C, followed by elution with LDS buffer (Thermo Fisher, NP0007) at 90°C for 10 minutes. Ten µg protein was used for input and all the immunoprecipitated protein was used for subsequent MS analysis. Input protein and immunoprecipitated protein were resolved by electrophoresis until the whole lane was about 1 cm long. Coomassie blue staining (Biorad, 1610436) was used to identify proteins on the gel before whole lanes were sent and cut for MS analysis in the Health Sciences Mass Spectrometry Core at University of Pittsburgh. In brief, after peptides were extracted from the gel matrix, the peptides were enriched, and salts and detergents removed and prepared for MS analysis.

RNA-Seq

RNA samples were extracted with Direct-zol RNA Microprep kit (Zymo Research, R2062) according to the manufacturer's protocol. Sequencing and partial analysis was performed at Novogene Corporation. DESeq2 was used for differential gene expression analysis. Data visualization and enrichment analysis was performed with the GSEA desktop application (5), and clusterProfiler (6).

ChIP-seq

ChIP-seq samples were prepared with a ChIP Assay Kit (Millipore, 17-295) according to the manufacturer's protocol. Briefly, GSCs were dissociated to single cells, followed by washing with PBS. Cells were then crosslinked by adding formaldehyde directly to the culture medium to a final concentration of 1%. After a 10-minute incubation at 37°C, glycine (at a final concentration of 125 mM) was added to quench the reaction for 5 minutes at room temperature. Fixed cells were lysed and sheared, followed by incubation with Anti-L-lactyl-lysine rabbit monoclonal antibody (Ab) (PTM Biolabs, PTM-1401RM). Antibody-chromatin complexes were eluted, reverse crosslinked, and treated with RNase A (Millipore, 70856), followed by proteinase K (Sigma, EO0491). Finally, DNA was purified by ChIP DNA Clean & Concentrator kit (Zymo Research, D5201). Sequencing was performed by Novogene Corporation.

TrimGalore (https://www.bioinformatics.babraham.ac.uk/projects/trim_galore/) was used to trim raw FASTQ files. Trimmed data were aligned to the hg19 human genome assembly using Bowtie2, followed by sorting and indexing BAM files with SamTools. ChIP signals were visualized using bigwig files generated by DeepTools. "ComputeMatrix," "plotHeatmap," and "plotProfile" functions wrapped in DeepTools were used to generate heat maps.

Animal studies

All animal experiments were conducted under a protocol approved by Institutional Animal Care and Use Committee performed in University of Pittsburgh. NSG (NOD.Cg-*Prkdc*^{scid} *Il2rg*^{tm1Wjl}/SzJ) mice (IMSR Cat# JAX:005557, RRID: IMSR_JAX:005557, The Jackson Laboratory, Bar Harbor, ME)

were used to assess GSC growth *in vivo*. Briefly, both male and female mice aged from 4 to 6 weeks were randomly distributed to each group and maintained in an SPF animal facility in 12-hour light/12-hour dark cycle at University of Pittsburgh. We implanted 2×10^4 cells into the right hemisphere with stereotactic coordinates (from the bregma: x = 2 mm, y = −1 mm, z = −3 mm). For drug treatment experiments, mice were treated with equal doses of: vehicle (PBS); NaLac (Sodium L-lactate, Sigma, L7022; 1 g/kg/day); DCA (Dichloroacetate, Sigma, D54702; 150 mg/kg/day); or DCA (150 mg/kg/day) + NaLac (1 g/kg/day), by intraperitoneal injection from day 7 until the endpoint of the experiment. In murine GBM experiments, C57BL/6 female mice (Jackson Laboratory) were used to assess the growth of CT2A mouse glioma cells *in vivo*. Intracranial xenografts were established by implanting 2×10^4 CT2A cells into the right hemisphere of 4 to 6-week-old female mice with the same stereotactic coordinates as above. In DCA and CD47 antibody treatment experiments, mice were treated with equal amounts of PBS (vehicle control) or DCA (Dichloroacetate, 150 mg/kg/day) every day by intraperitoneal injection. IgG (100 μg/mouse) antibody or CD 47 neutralization antibodies (100 μg/mouse) were given by intraperitoneal injection at days 7 and 14. For microglia depletion experiments, mice were injected intraperitoneally with PLX5622 (Selleck, S8874) dissolved in sterilized water using a final dose of 50 μg/g daily, while mice in the vehicle control group were injected with an equal volume of sterilized water. The antibodies used in mouse experiments are listed in Supplemental Table 3. All mice were monitored daily until neurologic deterioration was evident, at which time mice were sacrificed. Gait changes, hunched posture, and lethargy were defined as signs of neurologic decline.

Bioinformatic analysis using public datasets

For bulk RNA-Seq analysis based on the datasets of TCGA, CGGA, and Rembrandt, all expression data (normalized count value) and survival information were downloaded via Gliovis (http://gliovis.bioinfo.cnio.es/). ESTIMATE (https://ibl.mdanderson.org/estimate/) was used to analyze the relationship of *CBX3* expression and immune infiltration. For single cell RNA-Seq analysis, UMAP visualization of *CBX3* expression (GSE84465) was performed using Single Cell Expression Atlas (https://www.ebi.ac.uk/gxa/sc/home). PCA visualization of *CBX3* expression in GSC and non-GSC tumor cells was performed using data in the Broad Institute Single-Cell Portal (https://singlecell.broadinstitute.org/single_cell, SCP503). GSCs were characterized by high expression of stem-like markers, while non-GSC tumor cells (non-GSCs) were characterized by high expression of mature astrocytic markers (1).

Supplemental Tables

Supplemental Table 1. Molecular profiling of GSCs

Glioblastoma stem cell model designation	Patient age (years), sex	Transcriptional subtype	Tumor grade
GSC23	63 years, male	Classical	Recurrent glioblastoma
CW468	Unknown	Undefined, with classical features	Unknown
D456	Unknown	Unknown	Glioblastoma
3565	32 years, male	Undefined, with classical features	Glioblastoma (grade IV)
RKI	Unknown	Classical	Unknown

Supplemental Table 2. shRNA sequences used

shRNA	Target Gene	Target sequence	Catalog number (Sigma)
shCONT	None	-	SHC016
shCBX3.295	Human *CBX3*	CGACGTGTAGTGAATGGGAAA	TRCN0000021916
shCBX3.370	Human *CBX3*	GCGTTTCTTAACTCTCAGAAA	TRCN0000021917
shCbx3_1	Murine *Cbx3*	TTGACAGTAGTTGGGATATTT	TRCN0000341051
shCbx3_2	Murine *Cbx3*	TCTCGACCCTGAACGAATAAT	TRCN0000341052
shLDHA.919	Human *LDHA*	CCACCATGATTAAGGGTCTTT	TRCN0000026538
shLDHA.1041	Human *LDHA*	CGTTTGAAGAAGAGTGCAGAT	TRCN0000026554

Supplemental Table 3. Reagents and Resources used in these studies.

Antibodies	SOURCE	IDENTIFIER
Anti-L-Lactyllysine Rabbit pAb	PTMBIO	PTM-1401RM
Anti-L-Lactyllysine Rabbit mAb	PTMBIO	PTM-1401RM
Histone-H3 Polyclonal antibody	Proteintech	17168-1-AP
LDHA-Specific Polyclonal antibody	Proteintech	19987-1-AP
Sox2 antibody	Proteintech	66411-1-Ig
His-Tag Monoclonal antibody	Proteintech	66005-1-Ig
CBX3 Monoclonal antibody	Proteintech	66446-1-Ig
DYKDDDDK tag Polyclonal antibody (Binds to FLAG® tag epitope)	Proteintech	20543-1-AP
Alpha Tubulin Polyclonal antibody	Proteintech	11224-1-AP
CD4 (D2E6M) Rabbit mAb	Cell Signaling Technology	93518S
CD8α (D8A8Y) Rabbit mAb	Cell Signaling Technology	85336S
Nestin (10C2) Mouse mAb	Cell Signaling Technology	33475S
Phospho-Stat3 (Tyr705) (D3A7) XP® Rabbit mAb	Cell Signaling Technology	9145T
Stat3 (79D7) Rabbit mAb	Cell Signaling Technology	4904S
p300 (D8Z4E) Rabbit mAb	Cell Signaling Technology	86377S
APC anti-human CD47 Antibody	BioLegend	323123

FITC anti-human CD274 (B7-H1, PD-L1) Antibody	BioLegend	393605
PE anti-mouse/human CD11b Antibody	BioLegend	101208
FITC anti-mouse/human CD11b Antibody	BioLegend	101205
FITC anti-human CD147 Antibody	BioLegend	306204
FITC anti-mouse CD8a Antibody	BioLegend	100705
Alexa Fluor® 647 anti-mouse CD45 Antibody	BioLegend	160303
Pacific Blue™ anti-mouse CD3 Antibody	BioLegend	100213
PE anti-mouse IFN-γ Antibody	BioLegend	163503
APC anti-mouse MHC II Antibody	BioLegend	107613
PE Rat IgG2b	BioLegend	400607
Alexa Fluor® 647 Anti-Firefly Luciferase	Abcam	ab237252
FITC Mouse IgG1	BioLegend	400108
PerCP-eFluor™ 710-Tmem119	Thermo Fisher	46-6119-82
CD47 Antibody	Signalway Antibody	32461
Monoclonal ANTI-FLAG® M2 antibody produced in mouse	Sigma	F1804-200UG
Anti-Olig2 Antibody	Millipore	MABN50
InVivoMAb anti-mouse/human/rat CD47 (IAP)	Biox cell	BE0283
InVivoMAb mouse IgG1 isotype control	Biox cell	BE0083
CBX3 Antibody	Novus Biologicals	NB100-2418

Olig2 Antibody	Novus Biologicals	AF2418
CD4 Monoclonal Antibody	Invitrogen	14-0042-82

Supplemental Table 4. Primer sequences for selected genes.

Primers		
Gene name	Forward primer	Reverse primer
ENO1	TCTCTTCACCTCAAAAGGTCTCT	CCATGGGCTGTGGGTTCTAA
GLUT1	CACTGTCGTGTCGCTGTTTG	AAAGATGGCCACGATGCTCA
GLUT3	TCTCTGGGATCAATGCTGTGT	TTCTTCCTGCCCTTTCCACC
HK2	CCCCAGCACAAAGCAGTCG	AGAGATACTGGTCAACCTTCTGC
MYC	GGCTCCTGGCAAAAGGTCA	CTGCGTAGTTGTGCTGATGT
OLIG2	CCAGAGCCCGATGACCTTTTT	CACTGCCTCCTAGCTTGTCC
IL4	TTTGCTGCCTCCAAGAACAC	TGTCGAGCCGTTTCAGGAAT
IL10	GGGCACCCAGTCTGAGAA	GCAACCCAGGTAACCCTTAAAGT
IL13	TTGCACAGACCAAGGCCC	AGCTGTCAGGTTGATGCTCC
IFNG	TCGCTTCCCTGTTTTAGCTGC	TCGGTAACTGACTTGAATGTCCA
PTPRS	AAGCACTCCGATTCGAGGAG	ACTTCTCGAAGCTCTCGCAC
CSPG4	CTCCACTCAGCTCCCAGCTC	ACCGAAGAAGGAAGCCGC
SPRED1	GGCGACTTCTGACAACGATAAT	AGTGACGCTGCTTAGTCCAC
CD59	GCGCCGCCAGGTTCT	GACGGCTGTTTTGCAGTCAG

PROCR	ACTTCTCTTTTCCCTAGACTGC	AGTCTTTGGAGGCCATCTGAG
CBX3	CCACGCCGCGAACGTAATA	TTCAGGCTCTGCCTCTTCAA
ACTB	CATGTACGTTGCTATCCAGGC	CTCCTTAATGTCACGCACGAT
TUBA1A	TCGATATTGAGCGTCCAACCT	CAAAGGCACGTTTGGCATACA

Supplemental References

1. Richards LM, Whitley OKN, MacLeod G, Cavalli FMG, Coutinho FJ, Jaramillo JE, et al. Gradient of Developmental and Injury Response transcriptional states defines functional vulnerabilities underpinning glioblastoma heterogeneity. *Nat Cancer.* 2021;2(2):157-73.
2. Bao S, Wu Q, Sathornsumetee S, Hao Y, Li Z, Hjelmeland AB, et al. Stem cell-like glioma cells promote tumor angiogenesis through vascular endothelial growth factor. *Cancer Res.* 2006;66(16):7843-8.
3. Genin M, Clement F, Fattaccioli A, Raes M, and Michiels C. M1 and M2 macrophages derived from THP-1 cells differentially modulate the response of cancer cells to etoposide. *BMC Cancer.* 2015;15:577.
4. Orihuela R, McPherson CA, and Harry GJ. Microglial M1/M2 polarization and metabolic states. *Br J Pharmacol.* 2016;173(4):649-65.
5. Subramanian A, Tamayo P, Mootha VK, Mukherjee S, Ebert BL, Gillette MA, et al. Gene set enrichment analysis: a knowledge-based approach for interpreting genome-wide expression profiles. *Proc Natl Acad Sci U S A.* 2005;102(43):15545-50.
6. Yu G, Wang LG, Han Y, and He QY. clusterProfiler: an R package for comparing biological themes among gene clusters. *OMICS.* 2012;16(5):284-7.
