# Supplementary material for: Lactate reprograms glioblastoma immunity through CBX3-regulated histone lactylation
Source: J Clin Invest. 2024 Nov 15;134(22):e176851. doi: 10.1172/JCI176851 (PMC11563687; doi:10.1172/JCI176851)

Full unedited blot for Figure 1A

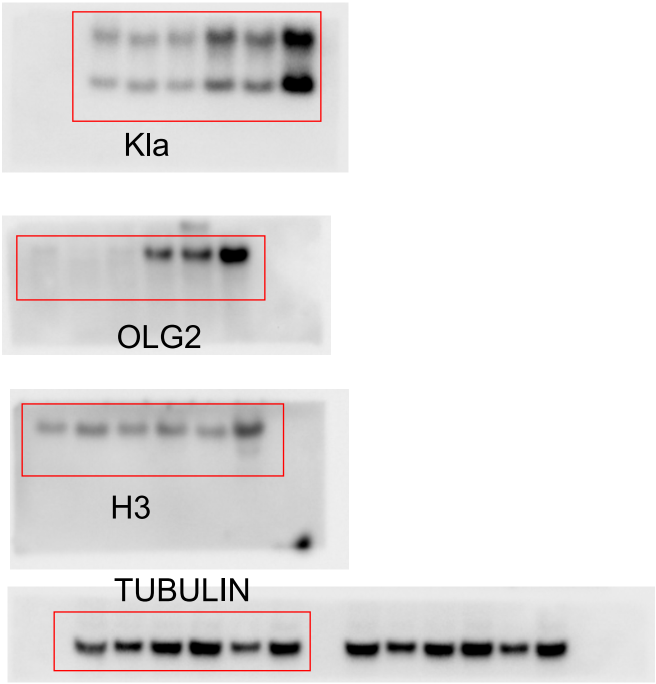

Full unedited blot for Figure 1D

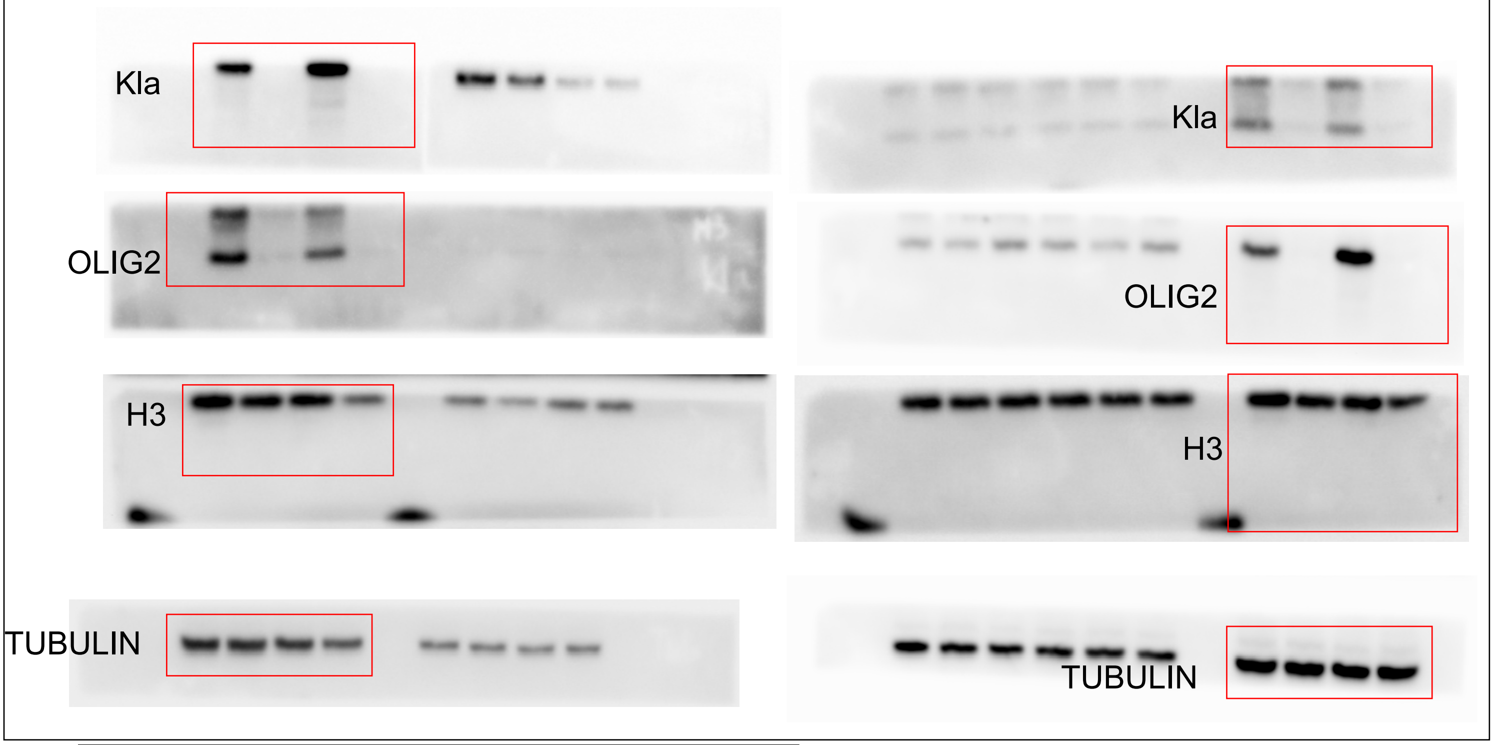

Full unedited blot for Figure 2G

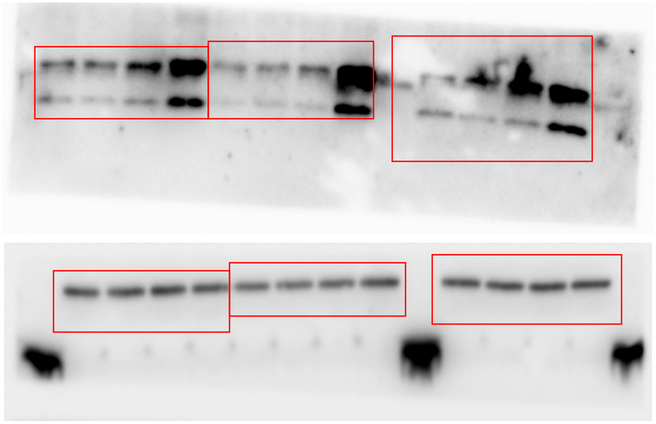

Full unedited blot for Figure 5A

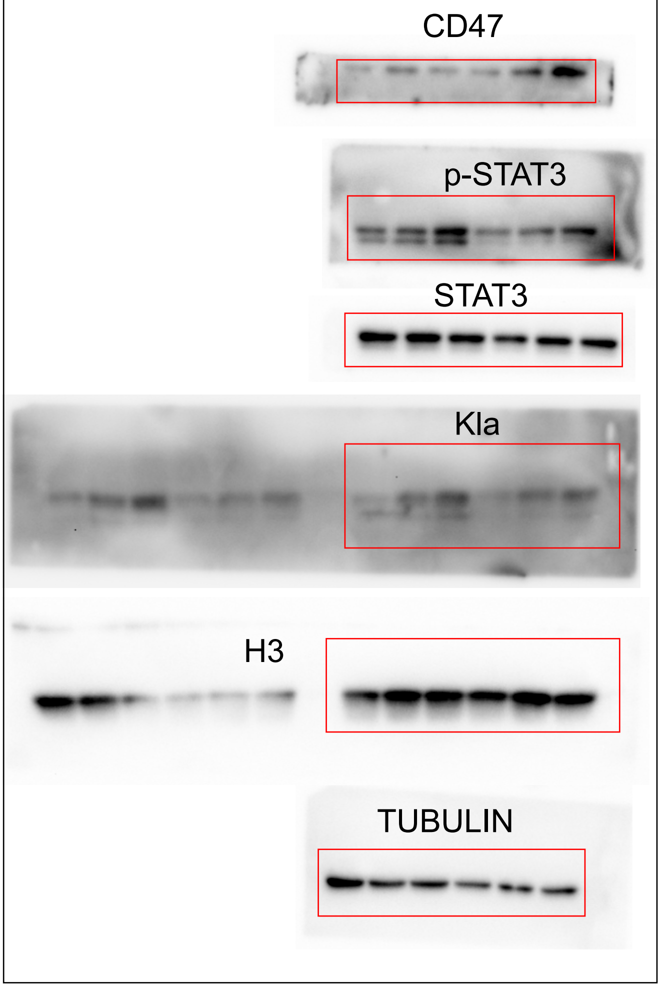

Full unedited blot for Figure 5C

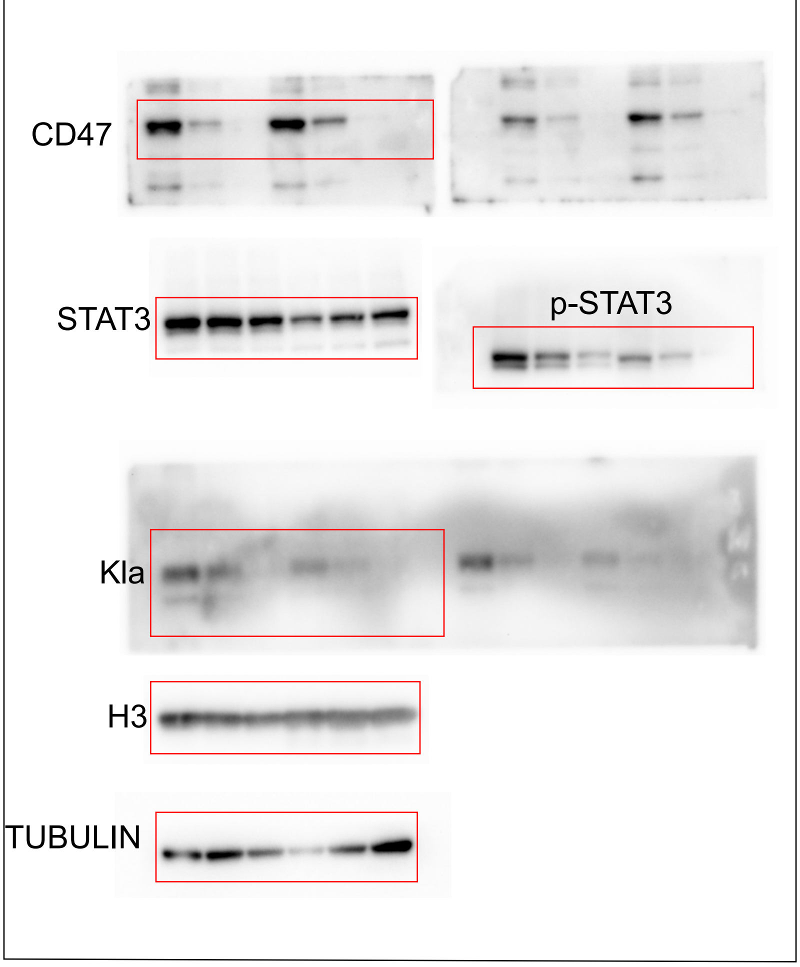

Full unedited blot for Figure 6C

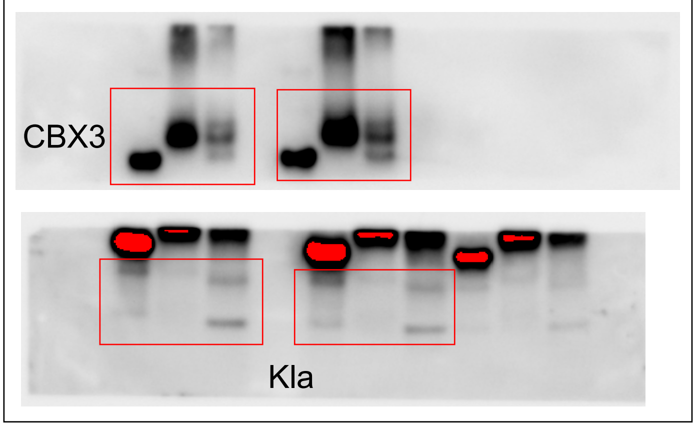

Full unedited blot for Figure 6E

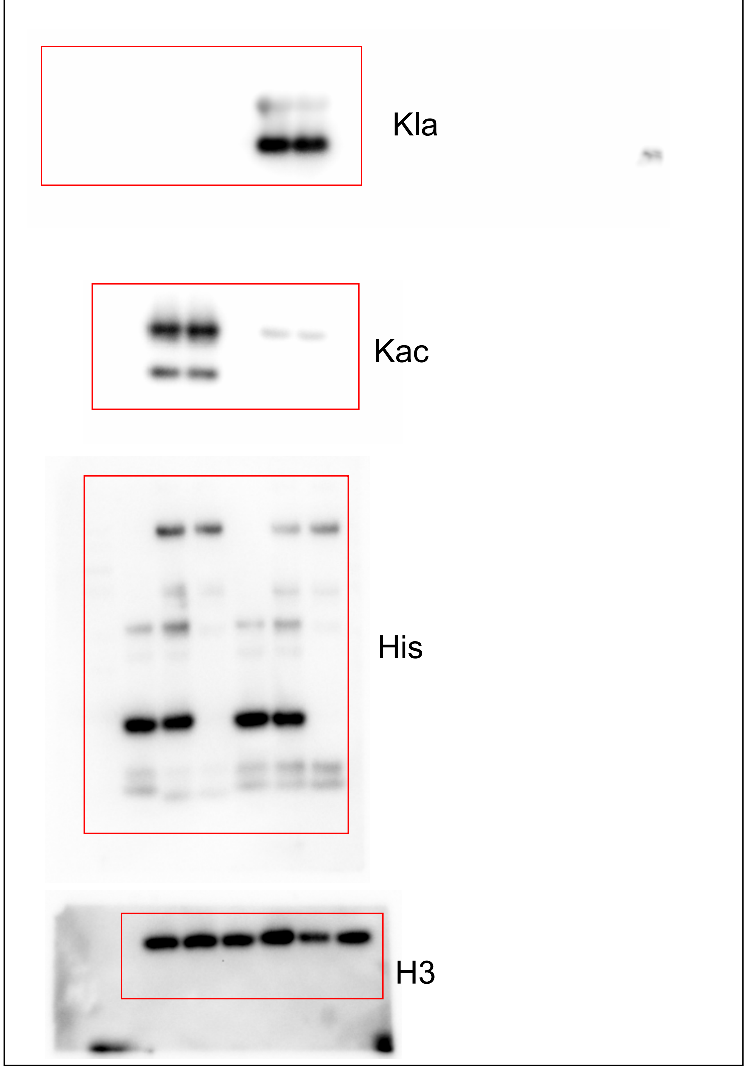

Full unedited blot for Figure 6F

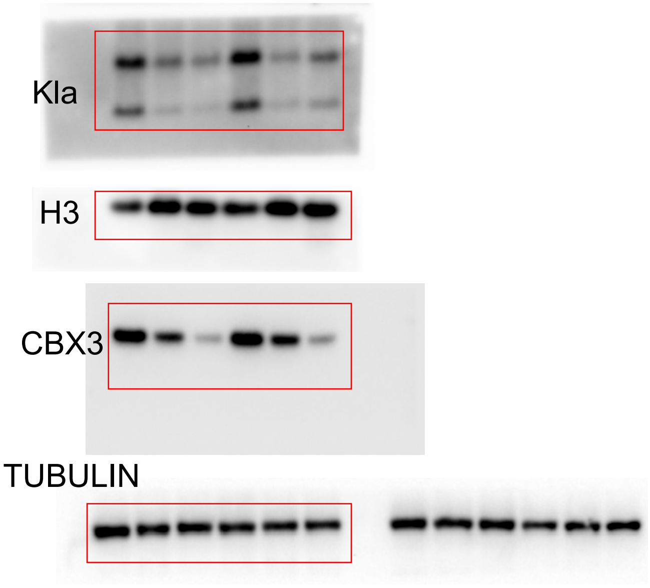

Full unedited blot for Figure 7D

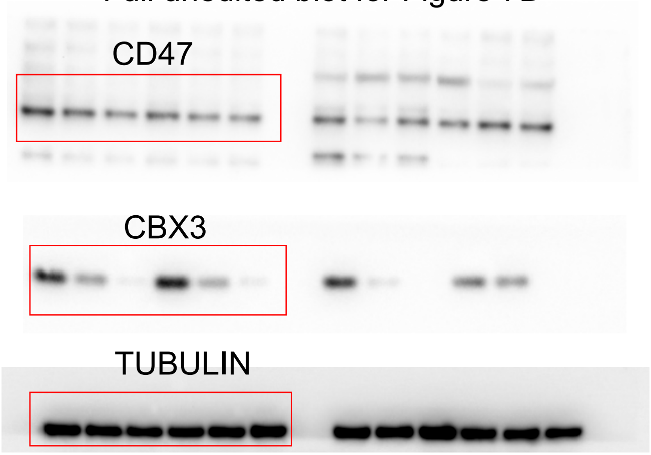

Full unedited blot for Figure 8E

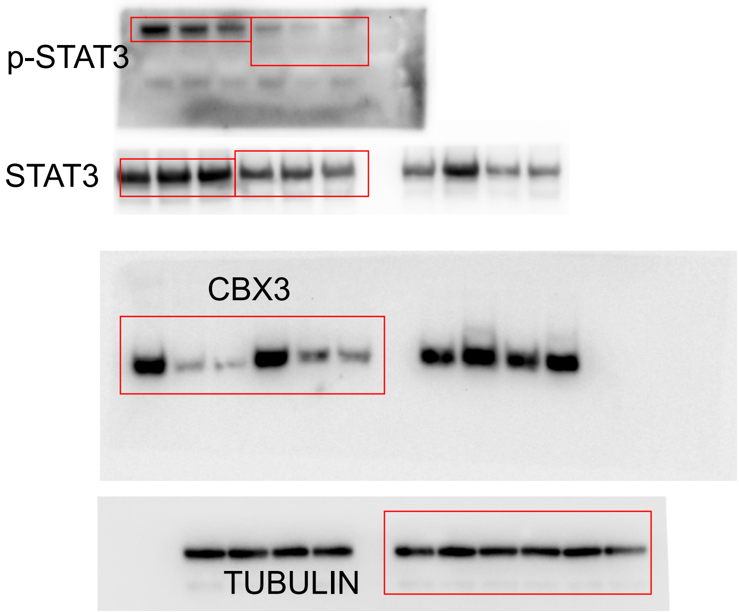

Full unedited blot for Figure 7E

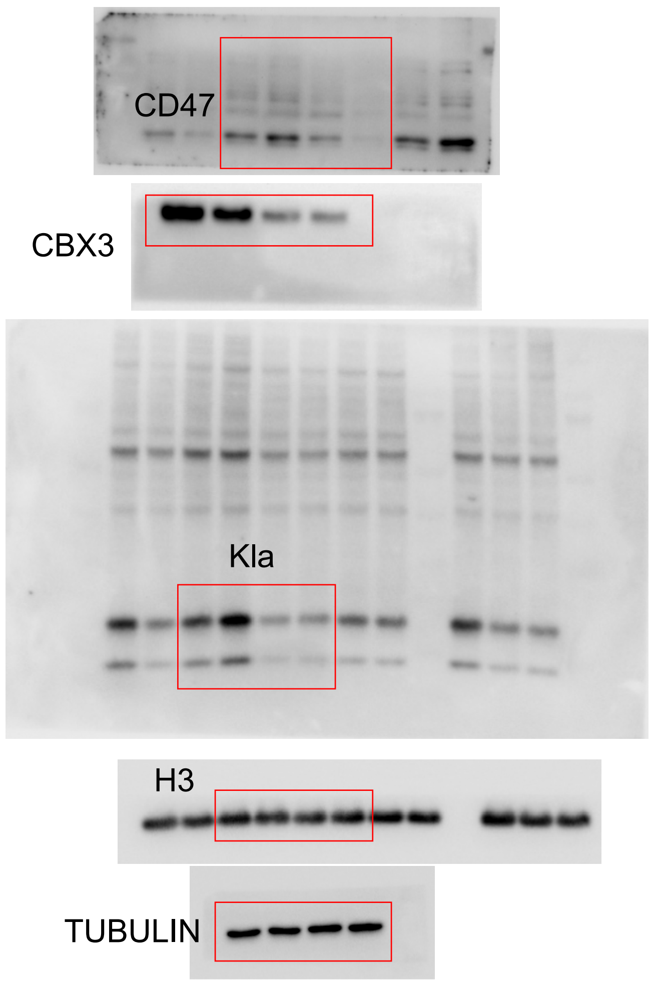

Full unedited blot for Figure S2G

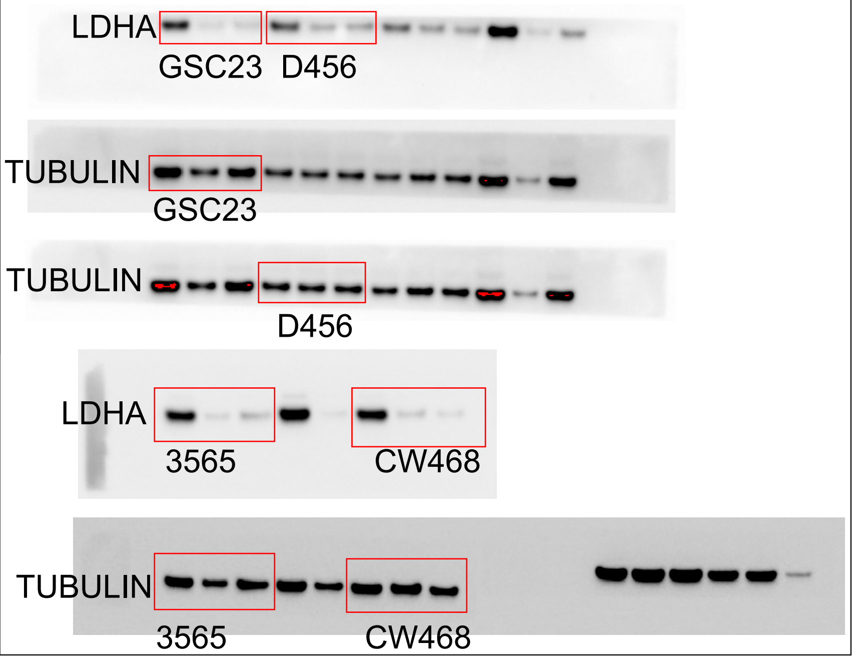

Full unedited blot for Figure S2P

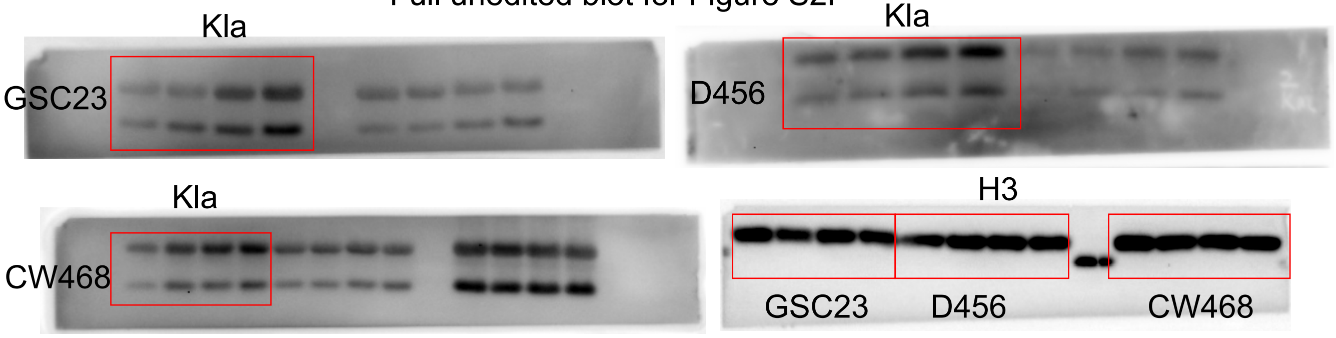

Full unedited blot for Figure S2S

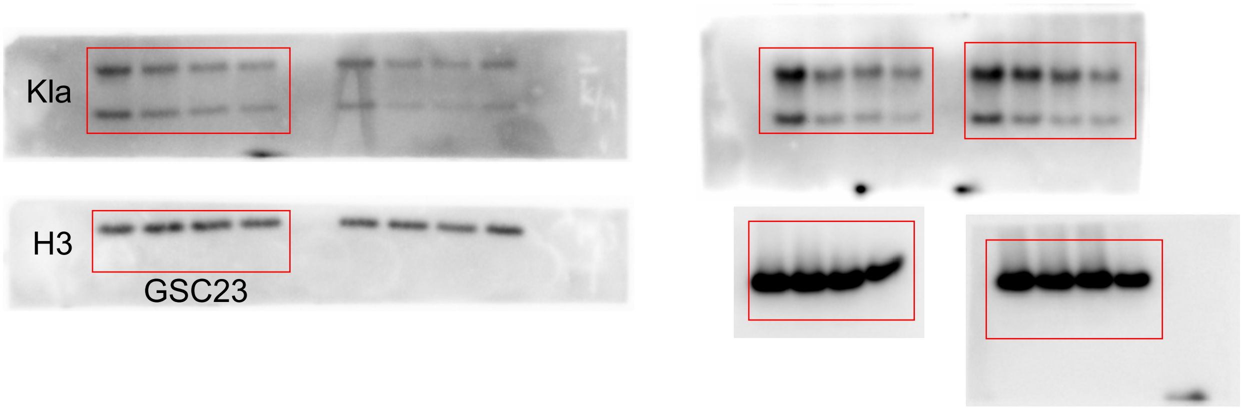

Full unedited blot for Figure S2U

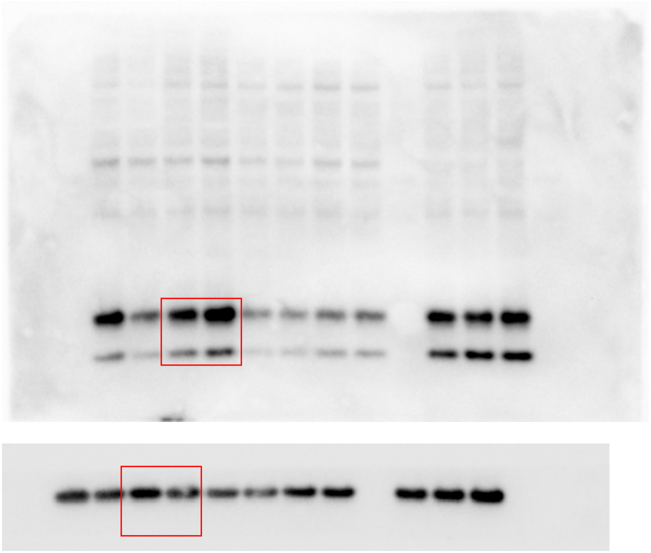

Full unedited blot for Figure S2V

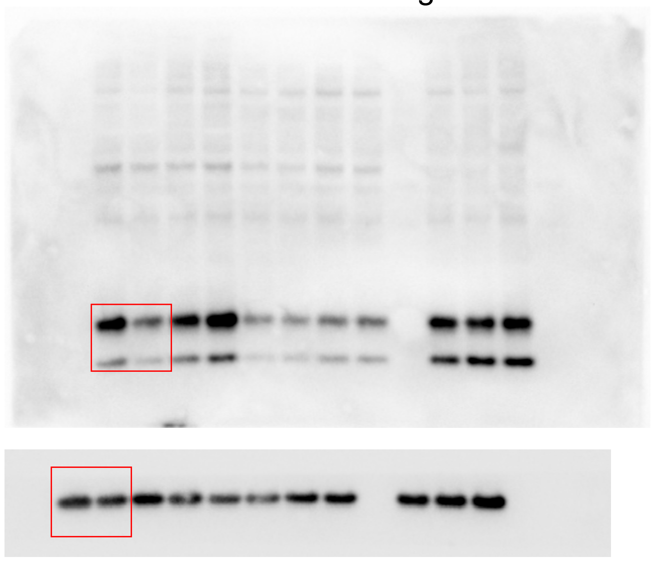

Full unedited blot for Figure S4B

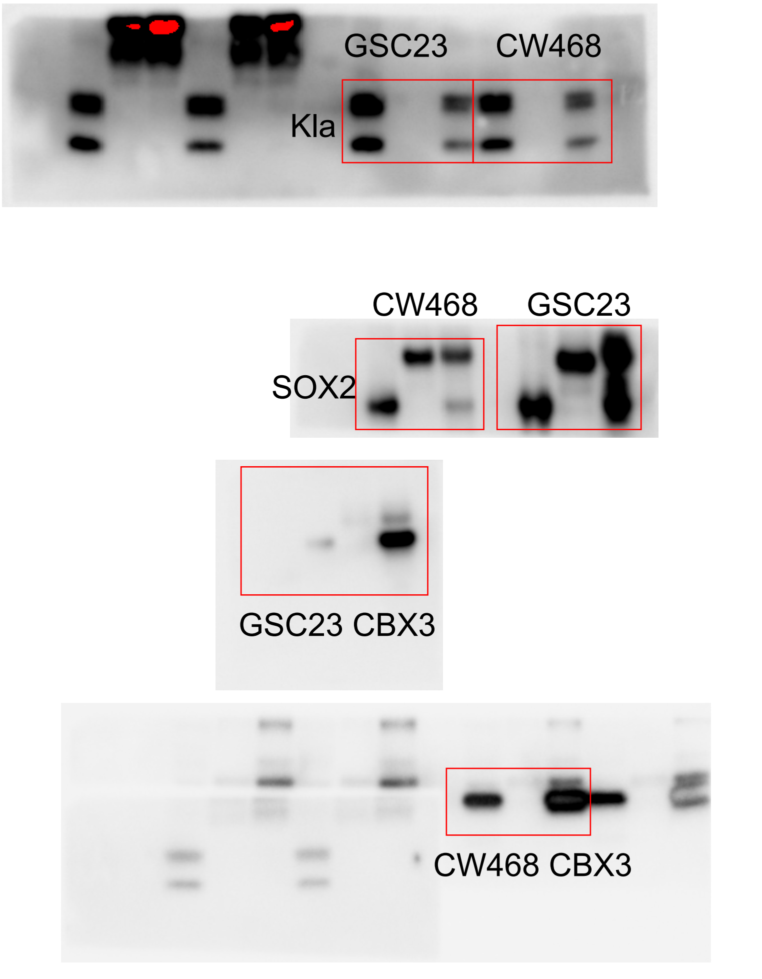

Full unedited blot for Figure S4C

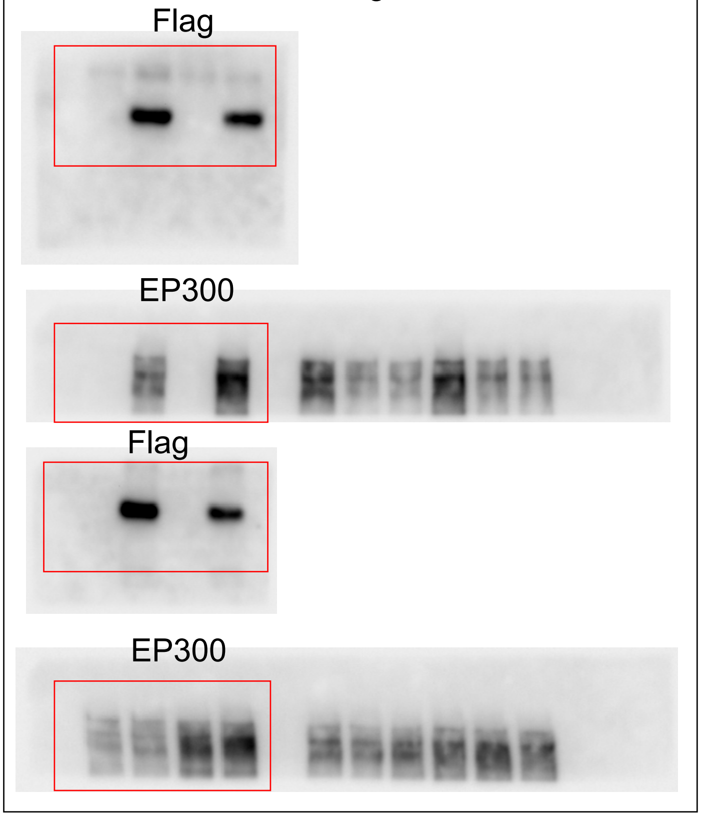

Full unedited blot for Figure S4D

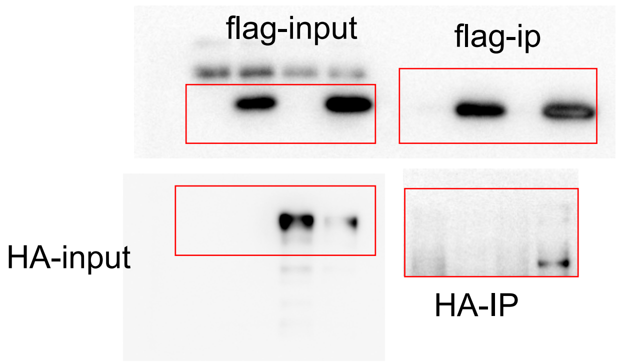

Full unedited blot for Figure S4I

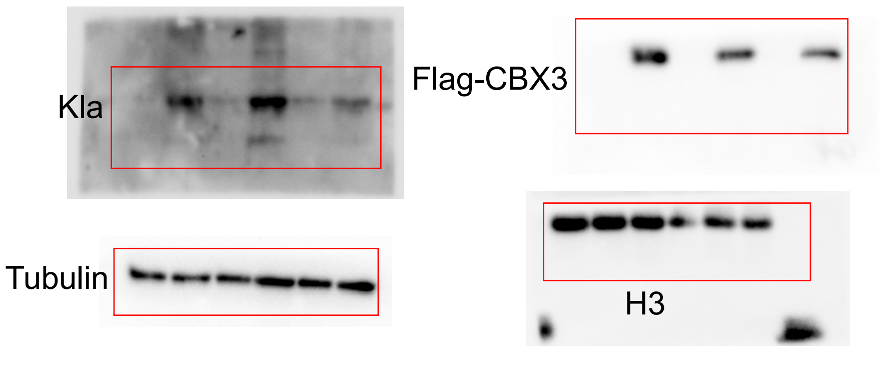

Full unedited blot for Figure S7D

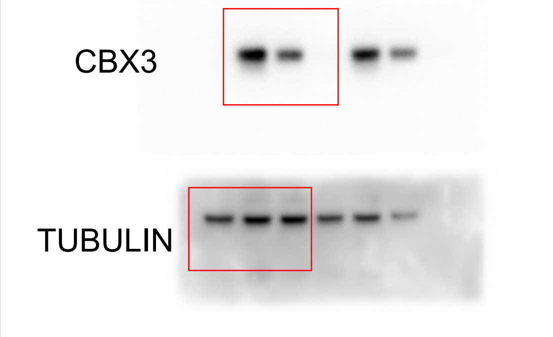

Supplement: Unedited blot and gel images [file jci-134-176851-s115.pdf]
